# Puerarin as a multifaceted anticancer agent: Mechanisms, targets, and therapeutic potential across multiple cancers

**DOI:** 10.1016/j.chmed.2025.11.001

**Published:** 2025-11-04

**Authors:** Aida Alizamir, Masoud Khanaghaei, Farshad Mirzavi, Ali Nokhodchi, Laleh Ghavami, Bita Taghizadeh

**Affiliations:** a Department of Pathology, School of Medicine, Hamadan University of Medical Sciences, Hamadan 6517838736, Iran; b Department of Laboratory Sciences, Sirjan Faculty of Medical Sciences, Sirjan 7816916338, Iran; c Cardiovascular Diseases Research Center, Birjand University of Medical Sciences, Birjand 9717853076, Iran; d School of Life Sciences, University of Sussex, Brighton BN1 9RH, UK; e Department of Biophysics, Institute of Biochemistry and Biophysics, University of Tehran, Tehran 1417614411, Iran; f Department of Medical Biotechnology, School of Advanced Medical Sciences, Tabriz University of Medical Sciences, Tabriz 5165665931, Iran

**Keywords:** chemosensitization, Gegen, isoflavone, Kudzu, multidrug resistance, *Pueraria lobata* (Wild.) Ohwi, tumor microenvironment

## Abstract

Puerarin (Pue), a key isoflavone from *Pueraria lobata*, has been traditionally used in Chinese medicine for cardiovascular, cerebrovascular, neurodegenerative, metabolic, and hepatic diseases. Recent studies indicate its anticancer potential across various malignancies. This review explores the current literature on Pue’s anticancer activities, mechanisms of action, and molecular targets. Preclinical *in vitro* and *in vivo* studies suggest Pue’s effectiveness; however, challenges including standardization, selectivity, safety, and delivery hinder clinical translation. Specifically, dose optimization and rigorous standardization are needed to fully elucidate Pue’s mechanisms, particularly in hormone-dependent cancers. The development of advanced drug delivery systems, such as nanosuspensions, nanoemulsions, and nanoparticles, is essential to improve Pue’s poor water solubility and stability, and to enhance targeted delivery. Studies have shown that Pue can inhibit cancer cell proliferation, induce apoptosis, and suppress metastasis in various cancer cell lines, including breast, colon, lung, and ovarian cancers. Pue’s anticancer mechanisms involve modulating multiple signaling pathways, including nuclear factor-κB (NF-κB), extracellular signal-regulated kinase (ERK), Janus kinase 2/signal transducer and activator of transcription 3 (JAK2/STAT3), and Wnt signaling (Wnt), and targeting key molecules such as Caspases, Bcl-2-associated X protein (BAX), B-cell lymphoma-2 (BCL-2), matrix metalloproteinases (MMPs), and dual-specificity phosphatase 1 (DUSP1). Pue has also been found to enhance the efficacy of conventional chemotherapeutic agents such as cisplatin and oxaliplatin, and to overcome drug resistance. Overall, Pue shows promise as a multifaceted anticancer agent, which merits further investigation. This review provides a comprehensive overview of Pue’s anticancer effects, emphasizing its potential to guide future cancer treatment strategies.

## 1 Introduction

Cancer is the second leading cause of death globally. According to the International Agency for Research on Cancer (IARC) in 2022, nearly 20 million new cancer cases and almost 9.7 million cancer-related deaths were reported worldwide. It is expected that these numbers will rise in the future, as by 2050, the number of cancer diagnoses and mortality cases is expected to double (Bray et al., 2024). Despite significant advancements in cancer treatment, including surgery, chemotherapy, and radiotherapy, the prognosis for many cancer types remains poor. These conventional therapies often face limitations due to drug resistance, severe side effects, and damage to healthy tissues. Consequently, there is an urgent need to develop novel, safe, and effective anticancer drugs.

Natural products, particularly those derived from plants, have long been recognized as a rich source of therapeutic compounds. Approximately 60% of currently used anticancer drugs are derived from natural sources (Newman & Cragg, 2012). These compounds often exhibit diverse pharmacological activities and relatively low toxicity, making them attractive candidates for cancer therapy.

**Fig. 1:**
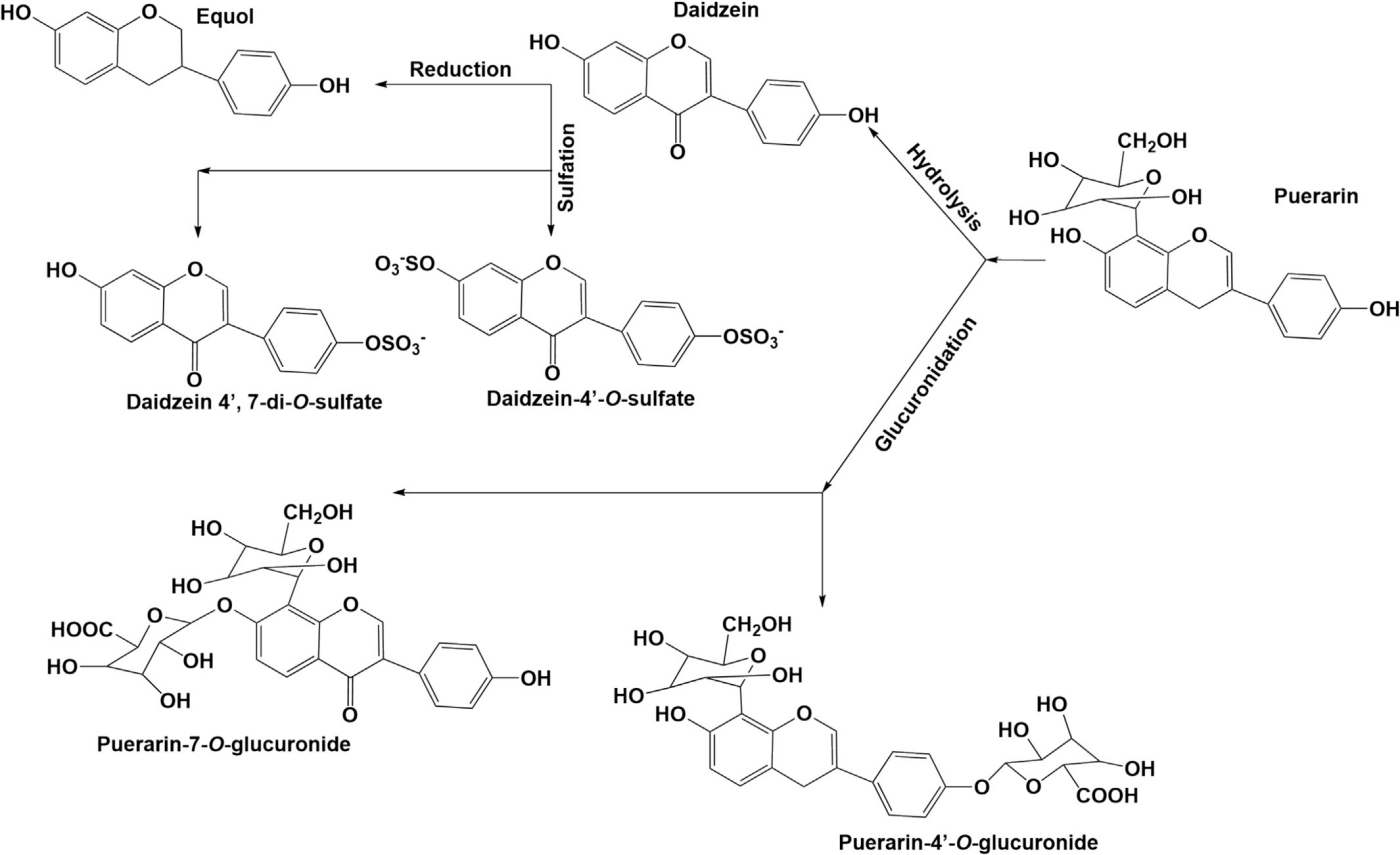
Pue and its metabolites.

*Pueraria lobata* (Wild.) Ohwi, known as Kudzu or Gegen, is widely distributed in East Asia. Its root has been used in traditional Chinese medicine for centuries to treat various conditions, including fever, diarrhea, cardiovascular diseases, alcoholism, diabetes (Liu et al., 2019, Qin et al., 2022, Xu et al., 2023, Yang et al., 2023, Yang et al., 2025, Zhang et al., 2013). Puerarin (Pue), the major isoflavone component extracted from *P. lobata*, has been shown to possess a wide range of pharmacological properties, including antioxidant, anti-inflammatory, and neuroprotective effects (Kulczyński et al., 2021, Liu et al., 2023, Zhang et al., 2023, Zhou et al., 2022, Zhou et al., 2021). Pue, chemically known as 7,4′-dihydroxyisoflavone-8*β*-glucopyranoside (molecular formula: C_21_H_20_O_9_, molecular weight: 416.38 g/mol), belongs to the isoflavonoid family. Pue’s structure (Fig. 1) comprises a basic isoflavone skeleton with hydroxyl and glucosyl substitutions. The presence of these functional groups contributes to Pue’s chemical properties and biological activities (Han et al., 2007, Malaivijitnond et al., 2010). Notably, Pue has also demonstrated significant anticancer potential against various types of cancer (Murahari, Singh, Chaubey, & Suvarna, 2020).

While previous reviews have explored the therapeutic potential of Pue, including its anticancer effects (Ahmad et al., 2020, Liga and Paul, 2024, Murahari et al., 2020, Peng et al., 2024), the present manuscript offers a distinct and comprehensive analysis by integrating the most recent research findings, including advancements in novel delivery systems and combination therapeutic strategies aimed at enhancing Pue’s anticancer efficacy. Additionally, this review addresses critical translational challenges, such as standardization, dose optimization, and safety evaluation, which are often underrepresented in earlier literature. By encompassing a broader and more current collection of studies across multiple cancer types and providing an in-depth mechanistic evaluation of Pue’s molecular targets and pathways, this review advances the existing knowledge, offering valuable insights to support the future clinical development of Pue as a versatile anticancer agent.

## 2 Pharmacokinetics, distribution, and absorption of Pue

The pharmacokinetics and biodistribution of Pue have been extensively investigated. Due to its chemical structure (Fig. 1), Pue has low water solubility (0.46 to 7.56 mg/mL) and intestinal permeability (Li et al., 2014, Quan et al., 2007), resulting in poor oral absorption, bioavailability, and limited clinical application. Intestinal absorption of Pue primarily occurs via the P-glycoprotein (P-gp) efflux transporter and multidrug resistance-associated proteins (MRPs) (Liang et al., 2012).

As shown in Fig. 1, Pue undergoes biotransformation through two principal pathways: functionalization and conjugation. The functionalization pathway involves the hydrolytic metabolism of Pue by the cytochrome P450 (CYP 450) enzyme in the liver, yielding daidzein. Daidzein can subsequently go through reduction, forming equol, or sulfation, resulting in the formation of daidzein-4′,7-di-*O*-sulfate, and daidzein-4′-*O*-sulfate (Prasain et al., 2009, Wen et al., 2008). The conjugation pathway involves UDP-glucuronosyl-transferase (UGT) in the liver, which catalyzes the formation of glucuronide conjugates, specifically puerarin-7-*O*-glucuronide and puerarin-4′-*O*-glucuronide (Anukunwithaya et al., 2018, Luo et al., 2010).

Anukunwithaya et al. studied the pharmacokinetic profile of Pue at dosages of 1, 5, and 10 mg/kg following both oral and intravenous (IV) administration in female rats (Anukunwithaya et al., 2018). Following oral administration, Pue reached peak plasma concentration within one hour, ranging from 140 to 230 μg/L. The absolute oral bioavailability of Pue was approximately 7% for the doses of 5 and 10 mg/kg. This limited oral bioavailability is attributed to Pue’s poor water solubility, significant first-pass metabolism, and limited intestinal permeability. The primary metabolites of Pue, daidzein, and the glucuronide conjugates exhibit enhanced water solubility, facilitating their excretion (Namken et al., 2021). Following IV administration, Pue demonstrated extensive distribution across various tissues, including the hippocampus, heart, lung, stomach, liver, mammary glands, kidney, spleen, femur, and tibia. Approximately 50% of the IV dose was excreted in the urine, with glucuronide conjugates identified as the predominant metabolites. These findings indicate that while Pue exhibits low oral bioavailability, it achieves widespread tissue distribution, including mammary tissues, upon IV administration (Anukunwithaya et al., 2018).

Furthermore, Pue was observed to cross the placenta and was detected in fetal plasma samples in rats, suggesting the necessity for cautious administration during pregnancy (Cao et al., 2013). To improve the oral absorption of Pue, various novel formulations have been developed, such as nano- and micro-emulsions, dendrimers, nanocrystals, and nanoparticle-based drug delivery systems (Zhang, 2019).

Namken and colleagues studied the absorption and distribution of pure Pue in comparison to Pue present within white kwao krua extract (PME) at the equivalent dose of 10 mg/kg in female cynomolgus monkeys (Namken et al., 2021). Blood, urine, and feces samples were collected and analyzed over seven d following oral administration. The results indicated that PME exhibited superior Pue absorption, bioavailability, and systemic concentrations compared to pure Pue, suggesting that the additional constituents within PME may enhance Pue's absorption and distribution. Peak plasma concentrations of Pue in monkeys were observed within two hours post-administration, followed by a gradual decline. Notably, the elimination of Pue was prolonged when administered as PME compared to the pure form (Namken et al., 2021).

Furthermore, Pue and its metabolites were detected in both urine and fecal samples of the monkeys, indicating excretion via these routes (Namken et al., 2021). PME showed enhanced absorption compared to pure Pue following a single oral dose. The oral bioavailability of Pue in monkeys was lower compared to rats, as reported by Anukunwithaya et al. The authors suggested that this difference could be attributed to more extensive first-pass metabolism in monkeys or higher intestinal membrane permeability in rats. The time to reach maximum concentration (T_max_), elimination half-life, and clearance of Pue following a single oral dose in monkeys closely resembled those observed in human studies (Anukunwithaya et al., 2018).

Kong et al. examined the pharmacokinetics and biodistribution of Pue in rats following intraperitoneal (IP) injection at varying concentrations (20, 40, and 80 mg/kg) (Kong et al., 2017). Pue concentrations were quantified in blood, heart, liver, spleen, lung, kidney, hippocampus, cerebral cortex, and striatum. At doses of 40 and 80 mg/kg, biphasic absorption was evident in blood and kidney samples, whereas other tissues exhibited a single absorption peak. The 20 mg/kg dose yielded only one peak in all analyzed tissues. Additionally, the area under the curve (AUC) and maximum concentration (*C_max_*) values exhibited a dose-dependent increase (Kong et al., 2017).

Penetar and colleagues investigated the pharmacokinetics of Pue following acute and repeated oral administration in human subjects after 8 and 72 h (Penetar et al., 2006). Pue was rapidly absorbed, reaching peak plasma concentrations at approximately 2 h, with an elimination half-life of approximately 4.3 h. Notably, the elimination half-life did not significantly change after repeated administration, suggesting consistent handling of Pue by the body over time. The study recommended a thrice-daily dosing regimen, as Pue did not exhibit accumulation, and its plasma levels remained within a biologically active range even 8 h after the last steady-state dose (Penetar et al., 2006).

Despite these promising preclinical and clinical findings, several pharmacokinetic challenges continue to limit Pue's clinical utility. Its low oral bioavailability, primarily due to limited solubility and extensive metabolism, underscores the need for parenteral delivery or advanced oral formulations that bypass or mitigate first-pass effects. Notably, the presence of co-occurring phytochemicals in herbal matrices such as PME appears to enhance systemic exposure to Pue, likely by modulating efflux transporters or metabolizing enzymes. This raises intriguing possibilities for synergistic combination strategies in formulation design. Furthermore, the widespread tissue distribution observed following IV administration, including high accumulation in mammary glands, may offer strategic advantages for targeting hormone-dependent cancers such as breast cancer. Lastly, the significant interspecies variation in pharmacokinetics highlights the importance of caution when extrapolating preclinical data and suggests the need for more advanced translational models.

## 3 Anticancer activity of Pue

### 3.1 Breast cancer

The initial investigation into the effect of Pue on breast cancer cells was conducted in 2003 by Boué et al. Their study examined the estrogenic activities of extracts from kudzu root and six other legumes (including soybean, red clover blossom, red clover sprout, alfalfa sprout, mung bean sprout, and green bean) on MCF-7 breast cancer cells (Boue et al., 2003). The Kudzu root extract exhibited significant estrogenic activity, competitive binding to the estrogen receptor (ER), and increased proliferation in MCF-7 cells. Notably, the kudzu root extract demonstrated the highest estrogenic activity and binding affinity for ER*α* (IC_50_ of 110 µg/mL) and ER*β* (IC_50_ of 22 µg/mL) in comparison to the other extracts. Utilizing high-performance liquid chromatography (HPLC), the researchers fractionated the kudzu root extract, identified Pue in fraction 4, and subsequently tested this Pue-containing fraction on MCF-7 cells. However, the precise concentration of Pue within this active fraction was not specified. The primary isoflavones identified in the kudzu root extract were Pue, daidzein, daidzin, genistein, and genistin, with Pue demonstrating the most pronounced stimulation of cell proliferation. The findings indicated a preferential agonistic activity of Pue toward ER*β*. Based on these observations, the authors suggested that Pue promotes the growth of estrogen-dependent breast cancer cells (Boue et al., 2003).

In contrast to the findings of Boué et al., Lin and colleagues reported that *Puerariae Radix* isoflavones (Pue, daidzein, and genistein) inhibited cell growth in both ER-positive (MCF-7) and triple-negative breast cancer cell lines (HS578T, MDA-MB-231) (Lin et al., 2009). Treatment with Pue at concentrations of 12.5, 25, 50, and 100 µmol/L resulted in growth inhibition, accompanied by Caspase-3-mediated apoptosis and cell cycle arrest at the G2/M phase in all three cell lines. Furthermore, the study revealed a significant increase in the active forms of Caspase-9 and Bax, as well as elevated expression levels of p53 and p21 (Lin et al., 2009).

The seemingly inconsistent findings reported by Boué et al. (Boue et al., 2003) and Lin et al. (Lin et al., 2009) regarding the effects of Pue on breast cancer proliferation can be reconciled by integrating insights from the work of Nimpao (Nimpao, 2006), which highlights the critical influence of variables such as isoflavonoid composition, environmental conditions, and metabolic activation. Boué et al.’s (Boue et al., 2003) observation of estrogenic activity and MCF-7 cell proliferation likely resulted from the use of a glycoside-rich *P. lobata* extract (containing compounds such as Pue, and daidzin) at unquantified concentrations. These glycosides may exhibit weak ER agonism in the absence of metabolic conversion to their more active aglycone forms. In contrast, Lin et al.’s demonstration of Pue’s antiproliferative effects likely reflects the use of purified aglycones (such as daidzein, and genistein) or metabolically activated Pue, which can effectively induce apoptosis. Nimpao further elucidated that clone-specific isoflavonoid profiles in *Pueraria mirifica* (PM-III *vs* PM-IV) and environmental factors (temperature and rainfall) significantly govern bioactivity. PM-III, with its higher aglycoside/glycoside ratio (3.43-fold), tends to promote antiproliferative effects, whereas PM-IV, with its dominance of glycosides, may support estrogenic activities. The observation that metabolic activation led to an 8.65–9.18-fold increase in MCF-7 proliferation underscores the necessity of glycoside-to-aglycone conversion for achieving therapeutic efficacy (Nimpao, 2006). Furthermore, dose-dependent variability (e.g., proliferative effects at low glycoside doses versus growth inhibition at high concentrations) and methodological differences (crude extracts versus purified compounds) likely contribute to the observed discrepancies. These findings emphasize the critical need for standardized extraction protocols, controlled environmental conditions during plant cultivation, and consideration of the metabolic context when evaluating and optimizing the anticancer potential of Pue while mitigating potential estrogenic risks (Nimpao, 2006).

A concentration of 0.1 µmol/L Pue has been shown to decrease matrix metalloproteinase 16 (MMP-16) expression and promote apoptosis in MCF-7 cells by inducing G2/M cell cycle arrest (Zhang, Wang, & Mo, 2018). Furthermore, Pue inhibited migration and metastasis in MCF-7 and MDA-MB-231 breast cancer cells through the downregulation of CCR7, CXCR4, MMP-2, MMP-9, intracellular adhesion molecule (ICAM), and vascular cell adhesion molecule (VCAM). Mechanistically, Pue treatment suppressed NF-κB and the ERK pathway, both of which are critical for cancer cell proliferation and growth (Liu, Zhao, Wang, Lin, & Yang, 2017).

In a study conducted by Hien and colleagues, Pue substantially downregulated the expression of *the multidrug-resistance 1 (MDR1)* gene, a key factor in drug resistance, in multidrug-resistant breast cancer cells (MCF-7/adr). This effect was mediated through the cAMP-responsive element (CRE)-dependent upregulation of AMPK and the downregulation of NF-κB. Pue treatment also enhanced the intracellular accumulation and cytotoxicity of adriamycin in these resistant cells (Hien, Kim, Han, Kang, & Jeong, 2010).

Zhifeng and colleagues investigated the potential of Pue to regulate the mRNA and protein expression of dual specificity phosphatase 1 (DUSP1) via miR-133a-3p expression in the ER-negative breast cancer cell line HCC38 (Li, Xu, Ren, Xu, & Chen, 2019). Their findings indicated that a 24-hour treatment of HCC38 cells with Pue resulted in a dose-dependent upregulation of miR-133a-3p. Both Pue and miR-133a-3p independently increased apoptosis in a time- and dose-dependent manner by upregulating the protein expression of Bax and cleaved Caspase-3 and 9. DUSP1 and p38 protein expression were significantly elevated in Pue-treated cells. Notably, Pue and mir-133a-3p exhibited synergistic effects in reducing cellular viability and promoting apoptosis. The authors concluded that Pue treatment increased miR-133a-3p expression, which subsequently led to increased expression of DUSP1 (Li et al., 2019). DUSP1 reduces cancer cell viability by downregulating the MAPK signaling pathway (Low & Zhang, 2016).

In a recent study by Chen et al., Pue enhanced the anticancer efficacy of oxaliplatin (OXA) and restored breast cancer's sensitivity to OXA both *in vitro* and *in vivo* (Chen et al., 2022). Pue promoted the anti-migratory effect of OXA and inhibited epithelial-mesenchymal transition (EMT) induced by low-dose OXA treatment in breast cancer by targeting carbonic anhydrase XII (Chen et al., 2022). Carbonic anhydrase XII is known to be actively involved in breast cancer migration and EMT, thereby promoting chemoresistance (Kopecka et al., 2015).

A recent investigation by Qiu et al. explored the anticancer effects of the Jin’gan capsules (JGCs) on MDA-MB-231, MDA-MB-468, and MCF-7 breast cancer cells (Qiu et al., 2022). JGCs, approved by the China Food and Drug Administration for the treatment of the common cold, were analyzed using UPLC-HR-MS/MS and network pharmacology, which identified cryptochlorogenic acid, neo-andrographolide, dehydroandrographolide, Pue, and harpagoside as the primary bioactive compounds. Treatment with JGCs demonstrated a dose-dependent anti-proliferative effect, particularly in the MDA-MB-231 and MDA-MB-468 cell lines. This effect was associated with cell cycle arrest, apoptosis, and downregulation of key proteins in the JAK2/STAT3 signaling pathway, suggesting that JGCs may suppress breast cancer cell proliferation (Qiu et al., 2022).

Through network pharmacology and analyses of the GEO database, Yu et al. identified 132 genes that were commonly altered in both breast cancer and mitochondrial disease datasets. Among these, 10 core genes were selected, including dystonin (DST), which was found to be downregulated in breast cancer. Molecular docking revealed a binding affinity between Pue and DST. Consistent with this, *in vitro* experiments demonstrated that Pue upregulated DST expression in a dose-dependent manner and inhibited proliferation and invasion in ER-negative HCC1806 breast cancer cells. Furthermore, Pue promoted apoptosis, as evidenced by increased Caspase-3 cleavage and Bax expression, and decreased Bcl-2 expression, which was accompanied by mitochondrial membrane potential disruption. *In vivo* studies showed that Pue significantly suppressed subcutaneous xenograft tumor growth and upregulated DST expression in breast tumor tissues. These findings suggest that DST represents a critical target of Pue in mitigating breast cancer progression through the induction of mitochondrial apoptosis and the inhibition of invasion (Yu, Li, & Xie, 2024).

**Fig. 2:**
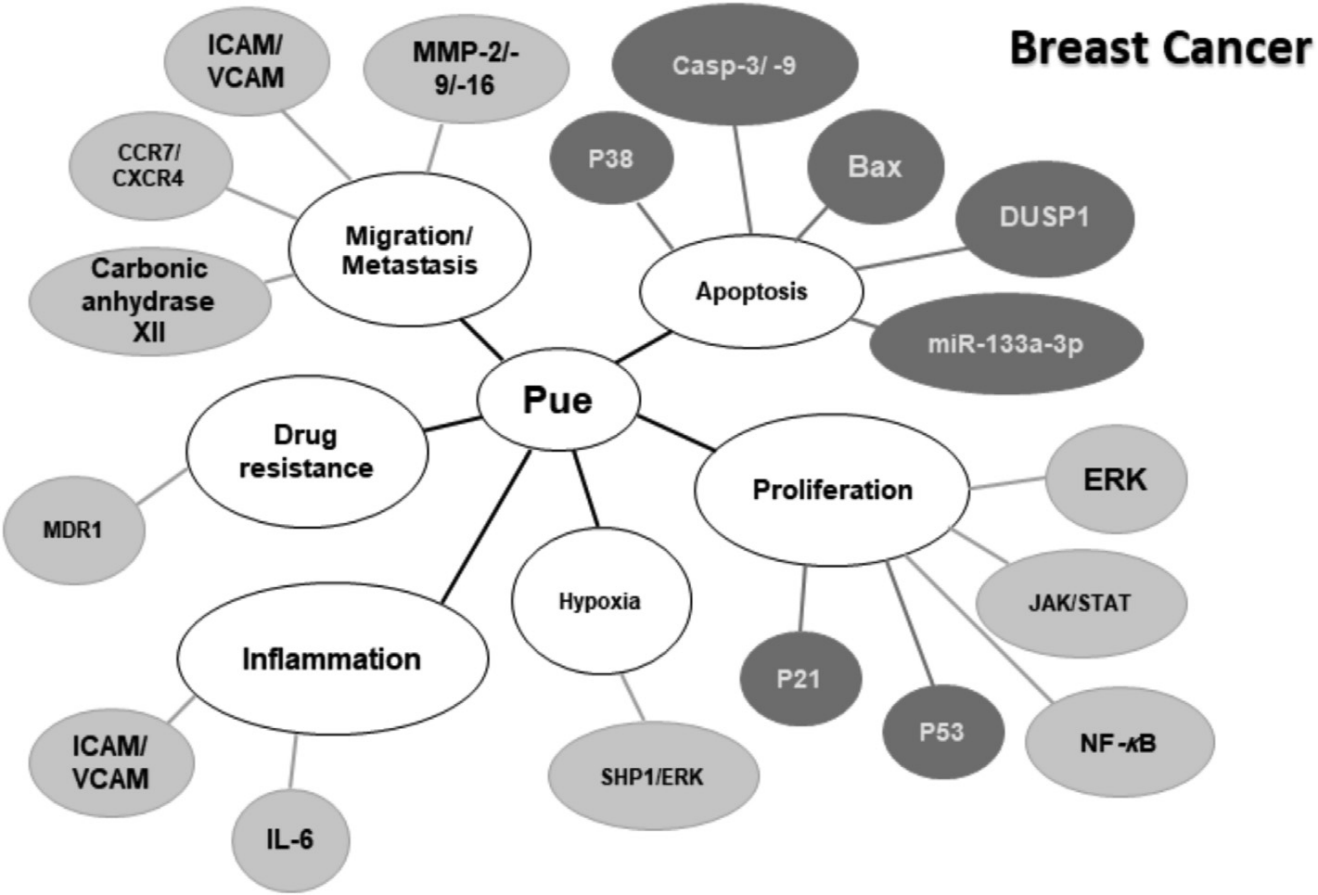
Pue targets in breast cancer. Light grey: inhibition, dark grey: promotion.

Fig. 2 illustrates the primary molecular targets of Pue in breast cancer. In summary, Pue has been shown to both inhibit cell proliferation and induce apoptosis and cell cycle arrest in ER-positive and ER-negative breast cancer cell lines. Furthermore, Pue reduces migration and metastasis by suppressing NF-κB signaling and downregulating MMPs. Pue has also been observed to upregulate miR-133a-3p, increase DUSP1 expression, and reduce breast cancer cell viability. Additionally, Pue demonstrates potential in overcoming chemotherapy resistance and augmenting immune responses in combination therapies by downregulating the MDR1 and improving the efficacy of adriamycin treatment.

### 3.2 Lung cancer

The effect of Pue on tumor-associated macrophages (TAMs) polarization and metastasis of non-small cell lung cancer (NSCLC) has been investigated (Kang et al., 2017). In murine models of NSCLC, Pue inhibited tumor growth and promoted the expression of M1 TAM markers (CD197, iNOS, CD40), which are associated with antitumor activity, while concurrently reducing the expression of M2 markers (CD206, Arg-1, CD163). Furthermore, Pue elevated the levels of anticancer cytokines (IFN-*γ*, TNF-*α*, IL-12), decreased tumor-promoting cytokines (IL-10, IL-4, TGF-*β*), and inhibited the invasion and migration of NSCLC cells by downregulating the expression of angiogenic factors (VEGF, MMP-9, and ICAM-1). Additionally, Pue inhibited the proliferation of NSCLC cells through the activation of the MEK/ERK1/2 pathway (Kang et al., 2017). Hu et al. also reported that Pue reduced the survival of NSCLC cells by suppressing cellular proliferation and inducing intrinsic apoptosis mediated by the mitochondria. Their *in vitro* results further demonstrated that Pue treatment induced autophagy via the activation of the PI3K/Akt and MAPK/ERK1/2 pathways. Consistent with *in vitro* findings, they also observed Pue-induced inhibition of tumor growth *in vivo* (Hu, Li, Lin, Liang, & Yan, 2018).

In 2020, Tao et al. investigated the effect of Puerarin 6″-*O*-xyloside (PRX) on lung cancer stem-like cells (LCSLCs) (Tao et al., 2020). LCSLCs, derived from the A549 lung adenocarcinoma cell line, are characterized by high self-renewal capacity, invasiveness, and tumorigenicity, along with the expression of stem cell markers (CD133, CD44, and ALDH1). Treatment with PRX reduced LCSLC viability, proliferation, and invasion by inhibiting the Akt/c-Myc pathway and decreasing stem cell marker expression (Tao et al., 2020). PRX also induced apoptosis in A549 cells through mitochondria-mediated pathways, as evidenced by the upregulation of Caspase-3, Caspase-7, Caspase-9, and Bax expression, and the downregulation of Bcl-2. Furthermore, PRX significantly inhibited tumor growth in a xenograft model of lung cancer when administered via IP injection at a dose of 40 mg/kg/day for 15 d (Chen, Chen, Wang, & Zhang, 2016).

Huang and colleagues demonstrated that Pue-induced apoptosis and the inhibition of cell growth, migration, and invasion in NSCLC are at least partially mediated through the upregulation of miR-342 and the subsequent downregulation of cyclin D1 expression, both *in vitro* and *in vivo* (Huang, Jin, Xu, & Wang, 2020). The dose-dependent suppression of cell growth, migration, and invasion by Pue has also been associated with the upregulation of miR-490 and the subsequent downregulation of denticleless E3 ubiquitin-protein ligase (DTL) in A549 cells *in vitro* (Zhang, Zhang, Zhao, & Zhao, 2022). The anti-invasive effects of Pue in lung cancer have also been linked to reduced expression of MMP-9 and MMP-2, key enzymes in tumor metastasis (Lang et al., 2022, Zeng et al., 2019). Furthermore, Pue treatment resulted in G0/G1 cell cycle arrest and a reduction in the S phase cell population in A549 cells *in vitro*. *In vivo* studies in lung cancer models showed that Pue decreased cyclooxygenase-2 (COX-2) enzymatic activity without affecting its protein expression. Additionally, Pue lowered prostaglandin E2 levels in A549 cells, a factor implicated in inflammation, lung cancer progression, and metastasis (Zeng et al., 2019).

Huang and Du investigated the potential of Pue to enhance the therapeutic effectiveness of cisplatin (DDP) against drug-resistant A549 lung cancer cells (A549/DDP). Their findings revealed a substantial decrease in cellular viability and survival when Pue and DDP were used in combination, compared to treatment with DDP alone. This combination therapy also resulted in reduced tumor growth and increased survival in a mouse model of A549/DDP lung cancer. The researchers concluded that Pue enhances the antitumor effect of DDP on drug-resistant A549 cancer cells by activating the Wnt signaling pathway (Huang & Du, 2020).

A recent study highlighted the potential of herbal medicines, particularly *Puerariae Radix* (PR) and its primary bioactive isoflavone, Pue, in the management of NSCLC, as illustrated by three reported cases. Pue played a critical role in Case 1 of the study, where a 79-year-old patient with metastatic EGFR/ALK-negative NSCLC achieved 19 months of progression-free survival (PFS) following the discontinuation of chemotherapy. Administered as a component of the herbal formula Galgeunhaegi-tang, Pue contributed to tumor stabilization, normalized serum carcinoembryonic antigen (CEA) levels, and alleviated symptoms such as fatigue, dyspnea, and constipation. These observed effects collectively support a role for Pue in suppressing tumor progression and improving clinical outcomes in this specific case (Lee et al., 2023).

**Fig. 3:**
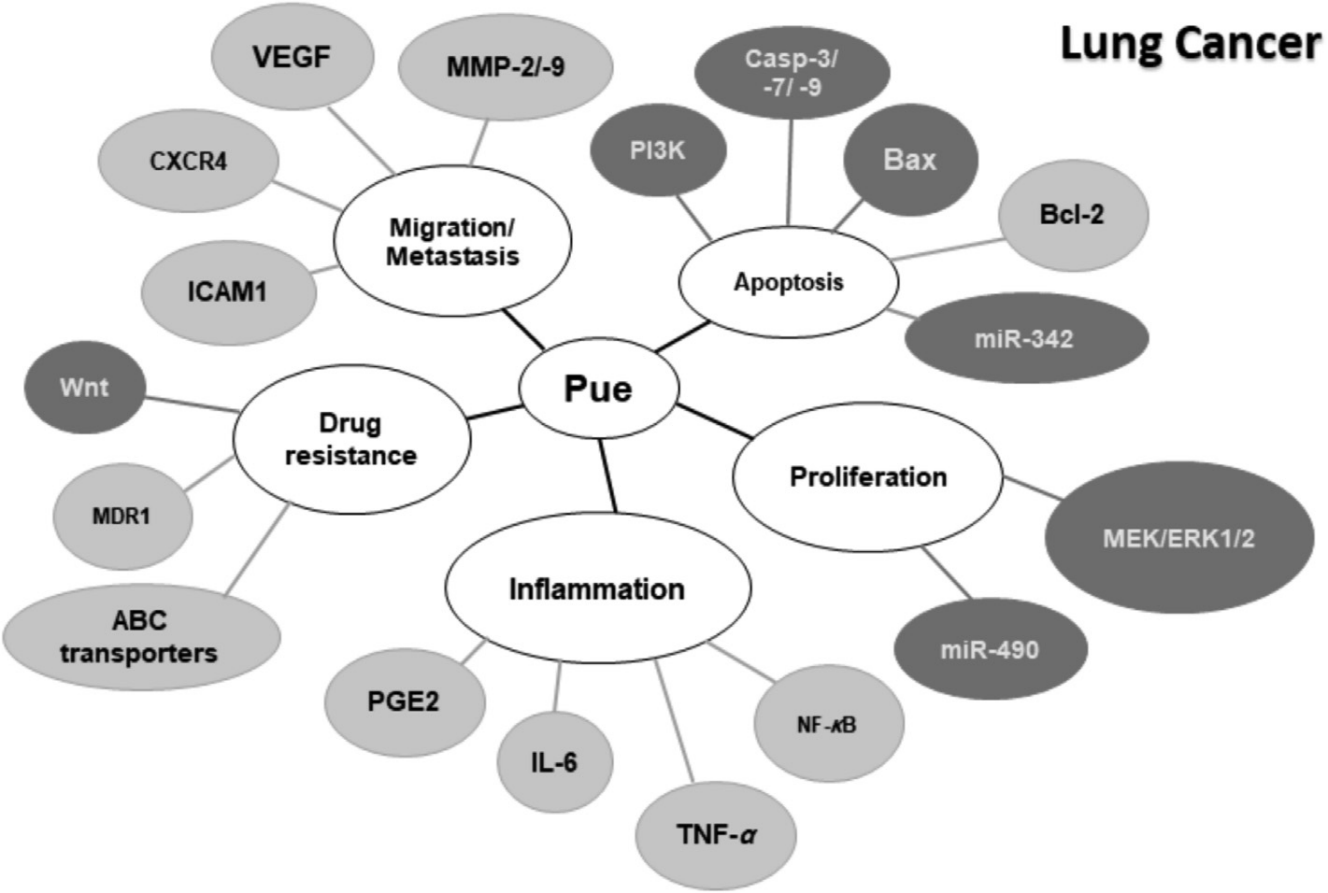
Pue targets in lung cancer. Light grey: inhibition, dark grey: promotion.

Fig. 3 presents the key molecular targets of Pue in lung cancer. In summary, Pue exerts anticancer effects against lung cancer through multiple mechanisms, including the inhibition of tumor growth, promotion of M1 TAMs polarization with a concomitant reduction in M2 markers, enhancement of anticancer cytokine production, and suppression of tumor-promoting cytokines. Pue also inhibited cancer cell invasion and migration by downregulating angiogenic factors and hindering cell proliferation via activation of the MEK/ERK1/2 pathway. Further investigations revealed Pue’s role in inducing apoptosis and activating autophagy through the PI3K/Akt and MAPK/ERK1/2 signaling pathways in lung cancer cells. Notably, Pue has been shown to enhance the therapeutic efficacy of DD in drug-resistant lung cancer models, leading to improved survival. Collectively, these findings highlight the promising potential of Pue as a therapeutic agent in the treatment of lung cancer.

### 3.3 Ovarian cancer

Pue has demonstrated antagonistic activity against estrogen-stimulated malignant behavior in ovarian cancer cells (Han, Yu, & Shen, 2009). *In vitro* treatment with Pue substantially suppressed the migration and invasion of HO-8910 cells. Furthermore, the combinatorial treatment of these cells with both Pue and estrogen resulted in a reduction of cancer cell invasion and migration compared to cells treated with estrogen alone (Han et al., 2007). Pue also inhibited proliferation, induced apoptosis, and upregulated miR-125b expressions in chemoresistant SKOV3 ovarian cancer cells. RNA interference-mediated suppression of miR-125b attenuated the expression of apoptotic proteins, indicating that miR-125b is critical for Pue’s pro-apoptotic effects and suggesting its involvement in modulating drug resistance. This positions miR-125b as a key molecular target for enhancing therapeutic efficacy in ovarian cancer (Zhang, Duan, Bao, & Fu, 2017).

In 2015, Song and colleagues conducted a study to identify potential poly (ADP-ribose) polymerase-1 (PARP-1) inhibitors, a promising target for ovarian cancer, from a library of 13 000 natural products using a combination of computer screening and experimental enzyme assay (Song, Li, Li, & Kan, 2015). Employing a stepwise strategy, they narrowed down, selected, and identified promising candidates exhibiting high potency against PARP-1. Among the tested compounds, four natural products, including Pue, phloretin, chlorogenic acid, and biochanin A, demonstrated significant inhibitory activities against the catalytic domain of PARP-1, comparable to that of the FDA-approved PARP-1 inhibitor, olaparib (Song et al., 2015). Notably, Pue exhibited a remarkable ability to inhibit the PARP-1 catalytic domain, with an IC_50_ of 6 nmol/L. The comparable potency of Pue to olaparib suggests its potential as a candidate for further development as a PARP-1 inhibitor in ovarian cancer therapeutics. Similar to other PARP-1 inhibitors, Pue interacted with the active site of PARP-1, which comprises a slender polar helix and a flat non-polar pocket. Pue formed strong chemical interactions by establishing hydrogen bonds and electrostatic forces with the helix and exhibiting favorable binding within the pocket (Song et al., 2015). In the same year, Guo et al. demonstrated that Pue inhibited cell proliferation in SKOV3 and OVCAR3 cell lines in a dose-dependent manner and promoted apoptosis by increasing miR-125b and reducing Bcl-2 expression in both cell lines (Guo et al., 2016). Furthermore, Duan et al. showed that Pue effectively suppressed the proliferation of cisplatin-resistant ovarian cancer cells by promoting apoptosis and restoring chemosensitivity. This effect was achieved through the inhibition of Silent mating type information regulation 2 homolog 1 (SIRT1) protein activity and blockade of the Wnt/*β*-catenin signaling pathway (Duan, Yin, Shao, Zheng, & Nie, 2021).

In a comprehensive study conducted by Ye and colleagues (Ye, Gao, Fang, Xu, & He, 2022), Pue significantly suppressed cell growth and induced apoptosis in the SKOV3 and NuTu-19 human ovarian cancer cell lines. The induction of apoptosis was accompanied by the overexpression of Bax and cleaved Caspase-3 and a reduction in the expression of Bcl-2. Furthermore, Pue treatment inhibited tumor formation in a rat model of NuTu-19 ovarian cancer through the activation of the PTEN and p53 tumor suppressor genes. Pue also reduced the elevated levels of IL-6, TNF-*α*, and CA125 (specific biomarkers of ovarian cancer) in the serum samples of rats. Notably, the researchers observed increased IP infiltration of CD8^+^ T lymphocytes following Pue administration. In addition, the study highlighted the modulatory effects of Pue on the gut microbiota, which has been implicated in tumor progression and immunosuppression in cancer. Interestingly, Pue was administered using two different protocols in this study: one group of animals received Pue at the initiation of ovarian cancer modeling, and another group received Pue after cancer modeling was established. Superior antitumor effects were observed in the first group, indicating the importance of the timing of Pue administration for achieving optimal therapeutic outcomes (Ye et al., 2022).

Lin et al. investigated the antitumor effects of *Pueraria montana* var. *lobata* (Willd.) on ovarian cancer in both *in vitro* and *in vivo* models (Lin, Su, & Ye, 2024). They identified seven active ingredients, including Pue, daidzin, daidzein, formononetin, ononin, and 3-methoxy Pue. Molecular docking studies revealed that Pue, the main active ingredient of *P. montana* var. *lobata*, exhibited a strong binding affinity to Caspase-3 and Jun. Based on these findings, the researchers proposed that Pue is responsible for the antitumor effects of *P. montana* var. *lobata*. *In vitro* experiments demonstrated increased protein levels of Caspase-3, Smac, and c-jun, supporting the initial molecular docking results. *In vivo* experiments further confirmed Pue's effectiveness in suppressing tumor growth and promoting apoptosis in tumor cells (Lin, Su, & Ye, 2024).

**Fig. 4:**
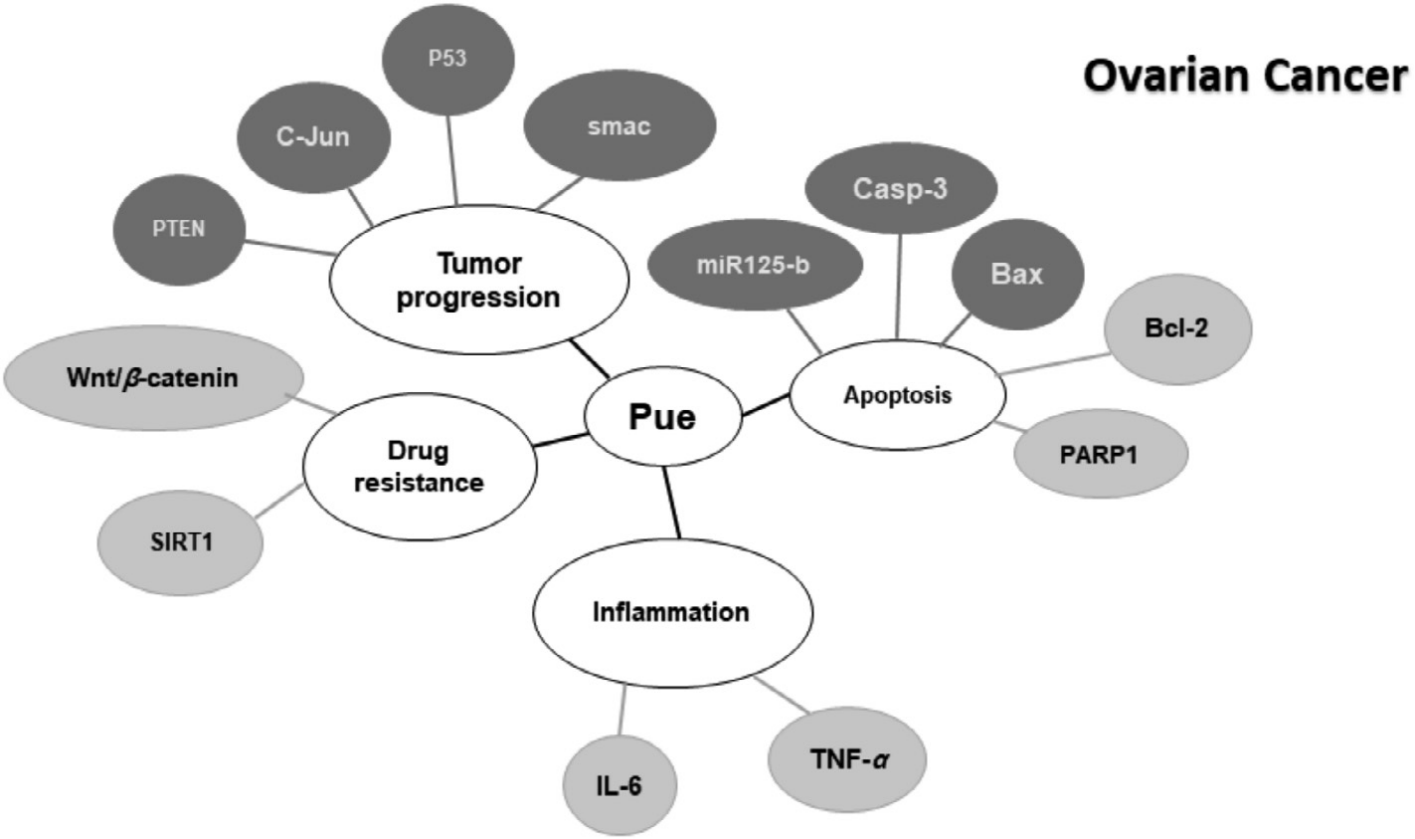
Pue targets in ovarian cancer. Light grey: inhibition, dark grey: promotion.

Fig. 4 summarizes Pue’s primary molecular targets in ovarian cancer. In summary, Pue has demonstrated antagonistic activity against estrogen stimulation, suppressing the migration and invasion of ovarian cancer cells. Pue has also been identified as a potent inhibitor of PARP-1, exhibiting comparable efficacy to olaparib. Furthermore, Pue has been shown to inhibit ovarian cancer cell proliferation, promote apoptosis, and restore chemosensitivity in cisplatin-resistant ovarian cancer cells by inhibiting SIRT1 and the Wnt/*β*-catenin signaling pathway. Pue also inhibited ovarian tumor growth and induced apoptosis by activating the tumor suppressor genes, PTEN, and p53. Additionally, Pue treatment increased CD8^+^ T lymphocyte infiltration, indicating an enhanced immune response. Overall, Pue demonstrates significant promise as a therapeutic agent for ovarian cancer treatment by inhibiting tumor growth, enhancing chemotherapy efficacy, and modulating immune responses.

### 3.4 Bladder cancer

Pue has been shown to inhibit cell proliferation, reduce cell survival, and induce apoptosis and cell cycle arrest at the G0/G1 phase in T24 and EJ bladder cancer cells *in vitro* (Jiang et al., 2018, Liu et al., 2018). Mechanistically, Pue downregulated the phosphorylation of mTOR and p70S6K proteins, while the total protein levels of these kinases remained unchanged, indicating that the suppression of cell growth by Pue is mediated through the inhibition of the mTOR/p70S6K signaling pathway. Furthermore, Pue prevented the invasion of bladder cancer cells *in vitro* (Jiang et al., 2018). Pue also downregulated NF-κB in T24 bladder cancer cells by increasing miR-16 expression and reducing COX-2 expression levels (Liu et al., 2018).

IYE and colleagues demonstrated that the induction of apoptosis following treatment of T24 cells with Pue was mediated through the upregulation of Bax and the downregulation of Bcl-2. In addition, both mRNA and protein expression levels of SIRT1 and p53 were substantially reduced. This finding suggests that the potential mechanism underlying Pue-induced apoptosis is related to the inhibition of the SIRT1/p53 signaling pathway in bladder cancer cells (Ye, Kan, Chen, & Lu, 2019).

Pue inhibited cell growth, migration, invasion, and metabolism of T24 and UM-UC-3 bladder cancer cells while promoting apoptosis via circ_0020394/miR-328-3p/nuclear receptor binding protein 1(NRBP1) axis (Du, Zhang, & Sun, 2022). Specifically, Pue decreased the expression of circ_0020394, which subsequently modulated the activity of miR-328-3p. MiR-328-3p directly targeted NRBP1, and its increased levels counteracted the pro-cancerous effects of circ_0020394. These findings were confirmed *in vivo*, demonstrating that Pue suppressed tumor growth by regulating the levels of circ_0020394, miR-328-3p, and NRBP1 (Du et al., 2022).

Recently, Ma et al. conducted comprehensive research on the impact of Pue on the T24 bladder cancer cell line (Ma et al., 2024). Consistent with prior studies, they observed a suppression of cellular proliferation in Pue-treated cells, with the IC_50_ of 218 µmol/L after a 48-hour incubation period. The authors performed in-depth bioinformatics analyses to identify the genes responsible for the anti-proliferative effect of Pue in these cells. They identified seven key targets involved in the growth and invasion of T24 cells: integrin subunit alpha 1 (ITGA1), laminin subunit alpha 3 (LAMA3), laminin subunit beta 3 (LAMB3), LAMA4, P21 activated kinase 2 (PAK2), Duchenne muscular dystrophy (DMD), and utrophin (UTRN). Their results indicated that Pue induced DNA damage in T24 cells *in vitro* and suggested that Pue-mediated inhibition of migration occurred through downregulation of ITGA1 gene expression (Ma et al., 2024).

Another recent study by Hao et al. demonstrated that Pue modulates centromere protein A (CENPA) to exert its anticancer effects against bladder cancer by targeting multiple molecular pathways. Key findings revealed that Pue downregulated CENPA expression, leading to reduced Aurora B kinase activity, a critical regulator of mitosis. This downregulation inhibited the PI3K/AKT/mTOR signaling pathway, impairing cell proliferation and survival. Pue also induced cell cycle arrest at the G_2_/M phase by modulating cyclin B1 and p21 and promoted apoptosis through the mitochondrial pathway via Bcl-2 downregulation, Bax upregulation, and Caspase-3 activation (Xu et al., 2024).

**Fig. 5:**
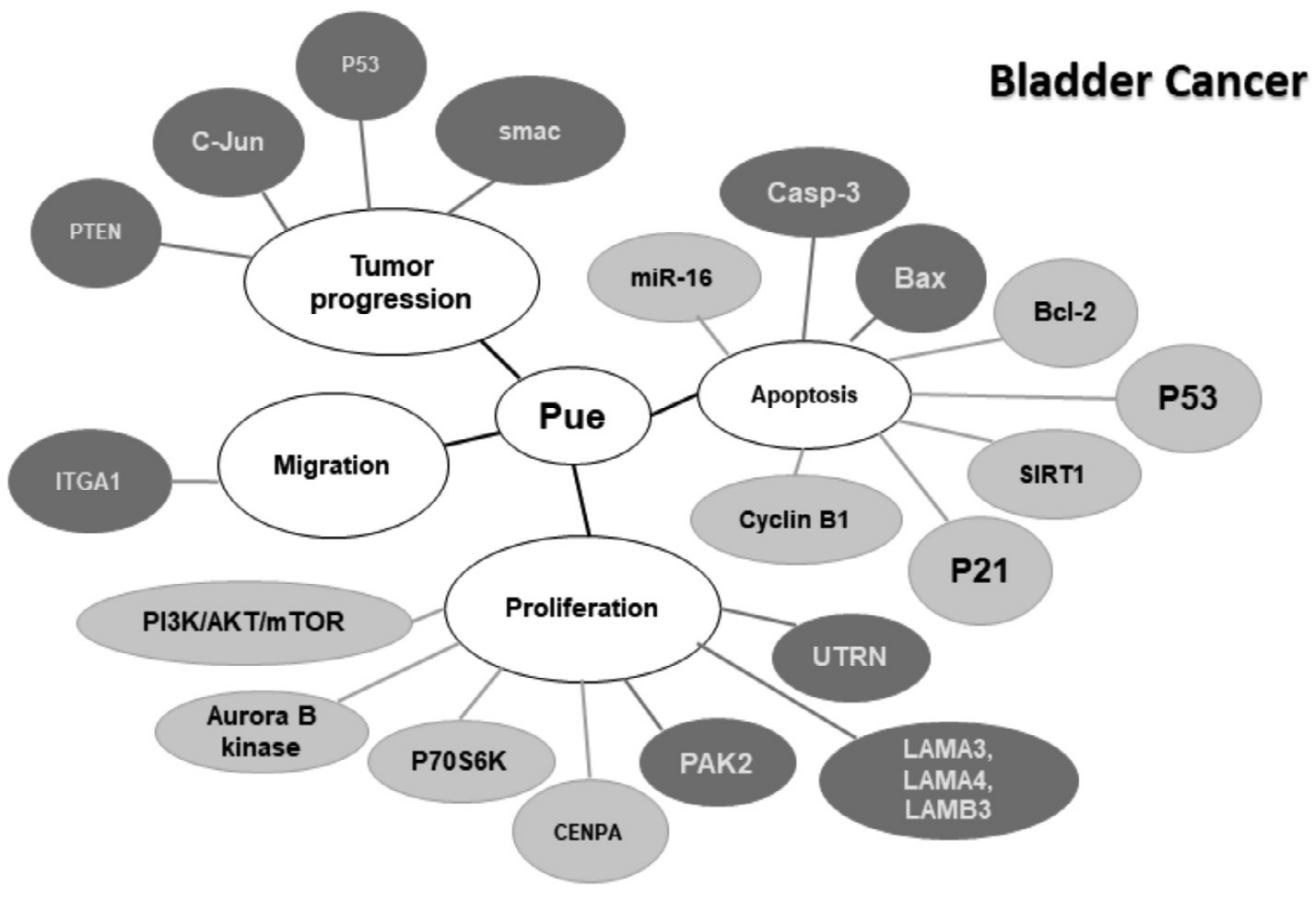
Pue targets in bladder cancer. Light grey: inhibition, dark grey: promotion.

Fig. 5 highlights Pue’s major molecular targets in bladder cancer. In summary, Pue has been shown to inhibit cell proliferation, induce apoptosis, and G_0_/G_1_ arrest in bladder cancer cells. The mTOR/p70S6K pathway mediates Pue's growth-suppressive effects. Additionally, Pue reduced bladder cancer invasion and inhibited the NF-κB signaling pathway by increasing miR-16 expression and decreasing COX-2 levels. Pue also inhibited cell growth, migration, invasion, and metabolism in T24 and UM-UC-3 cells by regulating the circ_0020394/ miR-328-3p/NRBP1 axis. Key genes associated with Pue's anti-proliferative effects in bladder cancer include ITGA1, ITGA1, LAMA3, LAMB3, LAMA4, PAK2, DMD, and UTRN. Furthermore, Pue modulates CENPA expression, leading to reduced Aurora B kinase activity and inhibition of the PI3K/AKT/mTOR pathway, as well as G_2_/M cell cycle arrest and mitochondrial apoptosis.

### 3.5 Prostate cancer

Li et al. demonstrated that Pue induced robust apoptosis in DU145, LNCaP and PC3 prostate cancer cells through upregulation of Bax and cleaved-Caspase-3, downregulation of Bcl-2, and a marked increase in ROS (Li, Xiong, Xu, Luo, & Zhang, 2021). Pue was more effective in inhibiting the proliferation of DU145 and PC3 cells compared to the LNCaP cell line, suggesting its superior toxicity towards androgen-independent cells. Notably, Pue showed no toxicity towards the normal epithelial prostate cell line PrEC. Concurrently, Pue treatment significantly elevated the secretion of pro-inflammatory cytokines IL-1*β* and IL-6 while markedly attenuating levels of the anti-inflammatory cytokine IL-10 (Li et al., 2021). The observed augmentation of ROS aligns with Pue’s capacity to disrupt redox homeostasis and trigger mitochondrial-mediated apoptosis in prostate cancer models. However, chronic elevation of IL-6 has been implicated in driving prostate cancer progression, therapeutic resistance, and metastatic dissemination via STAT3 signaling and androgen receptor activation (Chun et al., 2009, Smith et al., 2001), while enhanced IL-1*β* may promote angiogenesis, inflammatory stromal remodeling, and bone metastasis in advanced disease (DiNatale et al., 2022, Herroon et al., 2019). Conversely, the reduction of IL-10 (although potentially relieving immunosuppressive barriers) could impair anti-tumor immune surveillance and facilitate unchecked inflammation that paradoxically supports tumor growth (Shao et al., 2011, Yadav et al., 2021). Taken together, these *in vitro* findings underscore a dualistic immunomodulatory effect of Pue that, despite its pro-apoptotic efficacy, may inadvertently potentiate pro-tumorigenic cytokine networks *in vivo*. Future studies should therefore leverage relevant animal models to evaluate Pue’s net therapeutic index, incorporate longitudinal monitoring of systemic IL-6/IL-10 ratios, and assess combinatorial regimens with IL-6 pathway inhibitors or antioxidants to mitigate potential adverse inflammatory effects.

In another study, Pue demonstrated significant inhibitory effects on PC3 prostate cancer cells by suppressing proliferation, inducing apoptosis, and inhibiting migration and invasion. Pue downregulated phospho-Akt (p-Akt) and Bcl-2 expression, while upregulating Bax, Fas, Caspase-3, and Caspase-8, indicating Akt signaling inhibition and Caspase-mediated apoptosis induction (Pan et al., 2018).

**Fig. 6:**
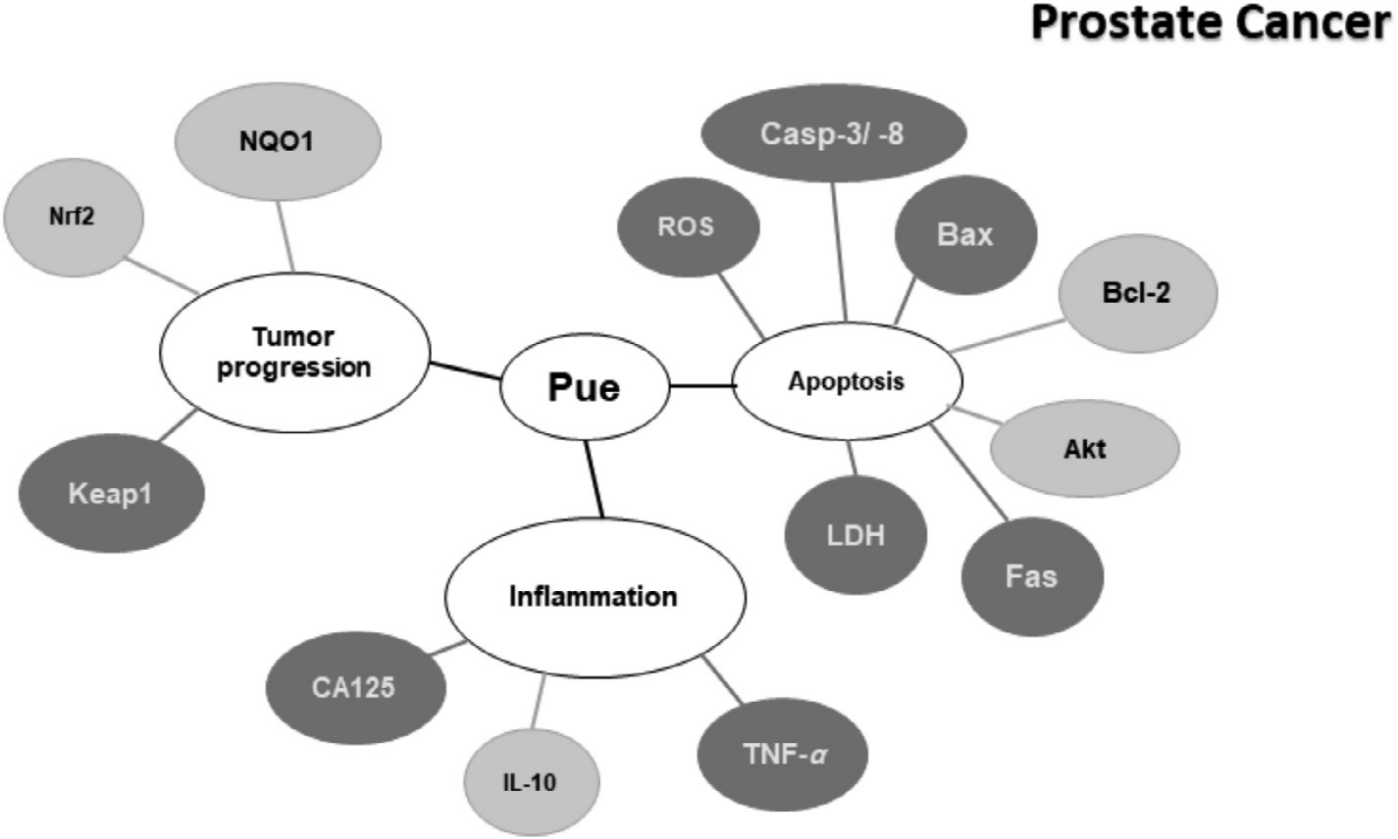
Pue targets in prostate cancer. Light grey: inhibition, dark grey: promotion.

As shown in Fig. 6, Pue acts on several key molecular targets in prostate cancer. In summary, TFP exhibited a biphasic effect, enhancing proliferation at low concentrations and inhibiting cell growth at high concentrations, while also demonstrating antioxidant activity by increasing SOD levels. Pue showed higher efficacy in inhibiting the proliferation of androgen-independent prostate cancer cells compared to androgen-dependent cells, without exhibiting toxicity towards normal prostate epithelial cells. Pue induced apoptosis in androgen-independent prostate cancer cells by modulating the expression of Bax, Bcl-2, and Caspase-3, Caspase-8, augmenting ROS, and LDH production, and altering the levels of pro- and anti-inflammatory cytokines. Additionally, Pue targeted the Keap1/Nrf2/ARE signaling pathway in these cells. Further research in PC3 prostate cancer cells demonstrated that Pue suppressed proliferation, and inhibited migration and invasion by downregulating p-Akt, indicating the involvement of Akt signaling inhibition.

### 3.6 Liver cancer

Pue inhibited SMMC-7721 cell growth and viability in a dose-dependent manner and increased the expression and phosphorylation levels of ERK1/2, JNK, and p38, indicating the involvement of the MAPK signaling pathway (Zhang et al., 2017). Pue also effectively suppressed hepatocellular carcinoma (HCC) growth, migration, and EMT by regulating Slug, Snail, miR-21, and PTEN (Zhou, Xue, Wang, & Ren, 2020).

The combinatorial treatment of HCC with Pue and 5FU has demonstrated superior antitumor potential compared to 5FU alone in the treatment of HCC via the induction of apoptosis and suppression of tumor growth (Zeng, Yang, Guo, Jun, & Dong, 2014). The inhibitory effect of Pue on HCC cell growth has shown to be both time- and dose-dependent (Zhang, Liu, Meng, & Hu, 2014). The induction of apoptosis by Pue involved ROS generation, disruption of the mitochondrial membrane potential, and increased expression of Caspase-3, Caspase-8, Caspase-9, and apoptosis-inducing factor (AIF) in HCC cells. Additionally, Pue increased the phosphorylation of p38, ERK1, and c-Jun N-terminal kinase (Zhang et al., 2014).

The anticancer mechanism of *Puerariae Lobatae Radix* against HCC was investigated using network pharmacology (Zhou et al., 2020). Network analysis identified Akt, IL6, MAPK3, EGFR, and PI3K/Akt pathway as the main targets of *Puerariae Lobatae Radix*. Molecular docking indicated that Pue exhibited high binding affinity to AKT1, MAPK3, MAPK1, and Caspase-3. Furthermore, Pue increased the phosphorylation of PTEN in human HCC cells through the PTEN/Akt/GSK3*β* signaling pathway, leading to decreased phosphorylation of downstream Akt (Zhou et al., 2020).

**Fig. 7:**
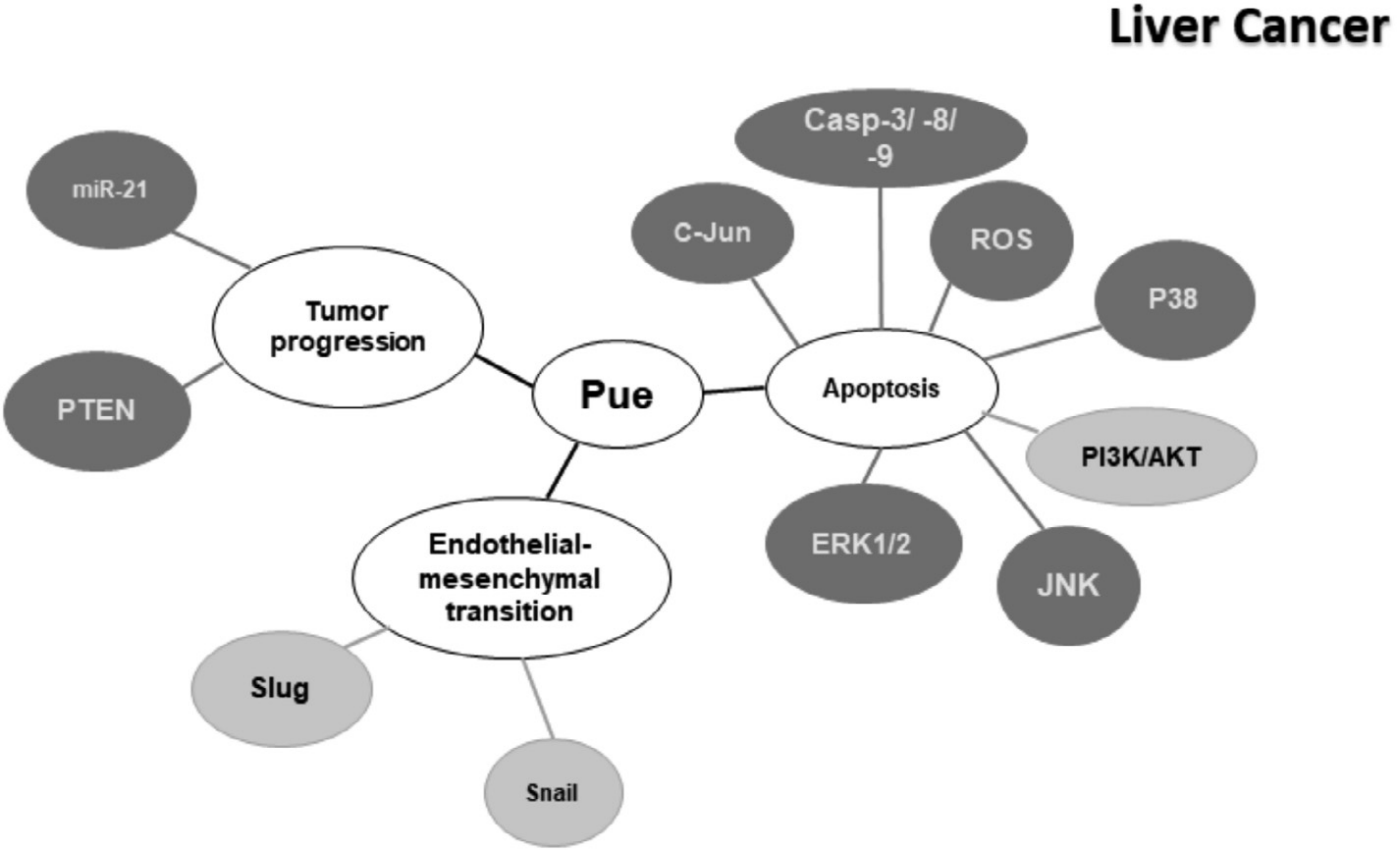
Pue targets in liver cancer. Light grey: inhibition, dark grey: promotion.

The main molecular targets of Pue in liver cancer are displayed in Fig. 7. In summary, Pue exhibits significant anticancer effects against HCC by inhibiting cell proliferation and activating the MAPK signaling pathway through increased ERK1/2, JNK, and p38 phosphorylation. Pue effectively suppressed migration and inhibited EMT by modulating key regulators such as Slug, Snail, miR-21, and PTEN. Moreover, Pue has demonstrated synergistic effects with 5-FU, enhancing its antitumor potential by inducing apoptosis and suppressing tumor growth in resistant HCC models. Apoptosis induction by Pue has been shown to involve ROS generation, mitochondrial membrane potential disruption, and increased expression of Caspases. Akt, IL6, MAPK3, EGFR, and PI3K/Akt pathway have been identified as key targets of Pue in HCC.

### 3.7 Other cancer types

Pue suppressed acute myeloid leukemia cell growth *in vitro* in a time- and dose-dependent manner, leading to decreased cell survival, which was accompanied by a reduction in the cellular population in the G0/G1 phase and an increase in the population in the S phase of the cell cycle (Shao et al., 2010). Combining Pue with arsenic trioxide has demonstrated synergistic anticancer effects on the acute myeloid leukemia cell line NB4 *in vitro*. Pue-induced apoptosis was mediated by inhibiting mRNA expression of Bcl-2, survivin, and promyelocytic leukemia/retinoic acid receptor alpha (PML/RAR*α*) genes. Concurrently, the protein expression levels of JNK, FasL, Caspase-3, and Caspase-8 were increased following Pue treatment (Tang et al., 2010). Autophagy has also been suggested as a mechanism of cell death induction by Pue in chronic myelogenous leukemia (Gao, Xiao, & Li, 2017).

Pue has shown therapeutic potential against mantle cell lymphoma (MCL) by inhibiting cell proliferation and inducing apoptosis via activating Caspase-3, -8, and -9 (Gan & Yin, 2015). Additionally, Pue downregulated the expression of various apoptotic proteins, including PARP, cyclin D1, Bax and Bcl-2, X-linked inhibitor of apoptosis protein (XIAP), and cellular inhibitor of apoptosis protein 1 (cIAP I), and inhibited PI3K/Akt and NF-κB signaling pathways, which are involved in cell survival and inflammation (Gan & Yin, 2015).

Yu and Li demonstrated that Pue effectively suppressed the growth of HT-29 colon cancer cells in a dose-dependent manner. Furthermore, Pue treatment induced apoptosis, accompanied by elevated levels of Bax and reduced levels of c-Myc and Bcl-2, as well as activation of Caspase-3 (Yu & Li, 2006). PRX inhibited cell growth in SW480, LoVo, and HCT-116 colon cancer cells with IC_50_ values of 37.1, 49.2, and 43 µg/mL, respectively. Apoptosis induction was mediated through increased Caspases-3 and -9 activity and decreased Bcl-2 expression. Moreover, reduced expression of MMP-3, MMP-9, and Vascular Endothelial Growth Factor (VEGF) proteins was observed, along with the inhibition of cancer cell invasion (Zhang et al., 2018).

A synergistic anticancer effect between Pue and 5FU has been reported in colorectal cancer (Guo et al., 2015). Network pharmacology analysis revealed that Pue exerts its anticancer effects via targeting tyrosyl-DNA phosphodiesterase-1 (TDP1), aldehyde dehydrogenase 1 family member A-1 (ALDH1A1), muscleblind-like splicing regulator 1 (MBNL1), aldehyde dehydrogenase-2 (ALDH2), and nicotinamide adenine dinucleotide (HPGD) in colorectal cancer (Li, Guo, Lu, & Tan, 2019). These targets are involved in energy pathways, metabolism, cell communication, signal transduction, aldehyde metabolism, and DNA repair. The antiproliferative effect of Pue on colon cancer cells has been linked to the induction of ferroptosis via nuclear receptor coactivator 4 (NCOA4) (Lian, Huang, & Zeng, 2023).

Pue also has demonstrated dose-dependent anti-proliferative and apoptotic effects on MGC-803 and AGS gastric cancer cell lines (Ma et al., 2013).

Oltulu et al. investigated the cytotoxicity of etoposide, alone or in combination with galangin or Pue on neuroblastoma and healthy astrocyte cell lines *in vitro*. Both Pue and galangin exhibited selective cytotoxicity toward neuroblastoma cells by enhancing the expression of topoisomerase 1 and 2*α*, Caspase-3, Caspase-9, Bax, Bcl-2, IL-1*β*, TNF*α*, and p53. Notably, Pue demonstrated superior cytotoxicity when used as a single agent compared to its combination with etoposide (Oltulu, Akinci, & Bakar, 2022). In a recent study, Sui et al. explored the therapeutic potential of Pue against neuroblastoma using bioinformatics and *in vitro* experiments (Sui, Liu, Zou, Zhang, & Zhang, 2024). Analysis of RNA sequencing data from neuroblastoma samples identified Baculoviral IAP repeat containing 5 (BIRC5), tissue inhibitor of metalloproteinases 2 (TIMP2), and Caspase-9 as prognostic genes. *In vitro* experiments on SH-SY5Y neuroblastoma cells revealed that Pue enhanced gap junction intercellular communication (GJIC) by upregulating connexin 43 (CX43) expression and reducing its phosphorylation. Pue also disrupted F-actin cytoskeleton organization, suppressing pseudopodia formation and motility, mediated by inhibiting the RhoA/ROCK1/MLCK signaling pathway. Gene and protein expression analyses confirmed that Pue downregulated the antiapoptotic protein BIRC5 and upregulated the metastasis inhibitor TIMP2 and Caspase-9. These findings underscore the multifaceted anticancer activity of Pue in neuroblastoma, targeting mitochondrial function, cytoskeletal integrity, and GJIC (Sui et al., 2024).

Concomitant treatment of Pue and 5FU has exhibited synergistic anticancer effects against esophageal cancer cells *in vitro* and *in vivo* (Wang et al., 2014). Pue treatment alone suppressed cell growth and induced apoptosis in these cells. Co-administration of Pue with 5FU enhanced the cytotoxic effects of 5FU on Eca-109 cells, as evidenced by increased apoptosis and growth inhibition (Wang et al., 2014).

Jia et al. investigated the impact of Pue on HPV 18-positive HeLa cervical cancer cells *in vitro* (Jia, Hu, Yang, & Li, 2019). Pue inhibited cell proliferation and promoted apoptosis by enhancing Caspase-3 and caspase-9 activity and the expression of Bax. In addition, Pue suppressed PI3K/Akt and mTOR expression in HPV-positive HeLa cells, suggesting its therapeutic potential in cervical cancer treatment through targeting the PI3K/Akt/mTOR signaling pathway (Jia et al., 2019).

Pue restored the sensitivity of CAL27 oral squamous cell carcinoma (OSCC) cells to cisplatin. CAL27 cells, modified to over-express or under-express the F-box and WD repeat domain containing 7 (FBXW7) tumor suppressor genes, were treated with cisplatin or Pue (Jiménez-Izquierdo et al., 2023, Shen et al., 2022). Over-expression of FBXW7 led to a significant decrease in HK2, PKM2, and LDH protein expression and suppressed the activity of hexokinase, pyruvate kinase, and lactate dehydrogenase. Additionally, FBXW7 over-expression reduced glucose uptake, lactate production, and decreased mTOR phosphorylation in a dose-dependent manner, thereby restoring cisplatin sensitivity. These results suggest that FBXW7 is a target for Pue in restoring cisplatin sensitivity in OSCC (Cai, Gao, Meng, & Chen, 2023).

The anticancer activities of Pue against highly invasive ductal adenocarcinoma of the pancreas were investigated by Zhu et al. (2021). Pue treatment markedly reduced cellular proliferation, migration, and invasion and induced apoptosis *in vitro*. Pue suppressed cellular migration via inhibiting EMT. In addition, Pue exhibited profound antitumor and anti-metastatic activities *in vivo* by inhibiting the Akt/mTOR pathway. Specifically, Pue deactivated the kinase domain of mTOR and inhibited ATP-Mg^2+^ attachment, consequently reducing glucose uptake and cellular metabolism (Zhu et al., 2021).

An *in silico* investigation was conducted by Ojo et al. to understand the interaction between Pue and specific cancer-related proteins (Ojo et al., 2022). In this study, the researchers identified five key proteins (CDK-2, Bcl-2, CDK-6, VEGFR, and IGF-1R) involved in tumor growth and investigated the inhibitory effects of Pue on these proteins. Pue exhibited superior binding to CDK-6 and Bcl-2 compared to doxorubicin, indicating its potential as an effective inhibitor of these proteins. Furthermore, the drug-likeness of Pue was confirmed using Veber’s rule, suggesting its suitability as a drug candidate (Ojo et al., 2022).

## 4 Systematic overview of signaling pathways modulated by Pue in cancer treatment

As detailed in the preceding section, Pue demonstrates a spectrum of anticancer activities *in vitro* and *in vivo* across various cancer types (Table 1), targeting several key molecular pathways and mechanisms. A comparative analysis of the presented information reveals that Pue consistently modulates specific signaling pathways involved in cancer progression (Table 2). This section systematically summarizes the molecular mechanisms and pathways regulated by Pue across various cancer types, based on the current preclinical literature.

**Table 1:** Pue administration protocol in *in vivo* tumor models.

Cancer type	Tumor model	Pue dosage	Duration of treatment	Method of administration	References
Breast	Murine	50 mg/kg	3 weeks	*po*	Chen et al., 2022, Xu et al., 2020
	Murine	35 mg/kg	19 d	iv	Liu et al., 2024
	Murine	30 mg/kg	9 d	Intratumoral injection	
Lung	Murine	40 mg/kg	30 d	iv	Kang et al., 2017
	Murine	25, 50 and 100 mg/kg	5 weeks	*po*	Hu et al., 2018
	Murine	40 mg/kg	15 d	iv	Chen et al., 2016
	Murine	60 mg/kg	7 d	iv	Xu et al., 2020
Ovarian	Muirne	30 mg/kg	23 d	Caudal vein injection	Duan et al., 2021
	Rat	40 mg/kg	3 and 9 weeks	ip	Ye et al., 2022
	Rat	40 mg/kg	3 and 9 weeks	ip	Lin et al., 2024
Bladder	Murine	100 µg/mL	5 weeks	Subcutaneous injection of Pue-treated UM-UC-3 cells	Du et al., 2022
Gastrointestinal and Colorectal	Murine	Free Pue (10 mg/kg) and Pue nanosuspension (2 and 10 mg/kg)	3 weeks	Tail vein injection	Wang et al., 2013
	Murine	Free Pue (8 mg/kg) and	3 weeks	ip	Deng et al., 2019
	Murine	Pue-loaded microspheres (4, 6 and 8 mg/kg)	3 weeks	−	Guo et al., 2015
	Murine	30 mg/kg	3 weeks	Tail vein injection	Yi et al., 2019
		5 mg/kg			
Hepatic	Murine	50 mg/kg	4 weeks	−	Zeng et al., 2014
Esophageal	Murine	25 mg/kg	3 weeks	−	Wang et al., 2014
Pancreatic	Murine	50 mg/kg	4 weeks	*po*	Zhu et al., 2021

**Table 2:** Pue’s anticancer activity and its targets in different cancer types.

Cancer type	Anticancer activity	Potential targets	References
Breast cancer	↓Proliferation	↑P53, ↑p21, ↓NF-*κ*B, ↓ERK, ↓JAK2/STAT3	Hien et al., 2010, Lin et al., 2009, Liu et al., 2017
	↑Apoptosis	↑Caspase-3, ↑Caspase-9, ↑Bax, ↑miR-133a-3p, ↑DUSP1, ↑p38, ↓Bcl-2	Li et al., 2019, Lin et al., 2009, Zhang et al., 2018
	Cell cycle arrest	G2/M	Lin et al., 2009, Zhang et al., 2018
	↓Invasion/metastasis	↓CCR7, ↓CXCR4, ↓MMP-2, ↓MMP-9, ↓MMP-16 ↓ICAM, ↓VCAM	Liu et al., 2017
	↓EMT	↓Carbonic anhydrase XII	Chen et al., 2022
	↓Drug resistance	↓MDR1, ↓ROS	Hien et al., 2010, Xu et al., 2020
	↓Inflammation	↓TNF-*α*, ↓IL-6	Liu et al., 2017
	↓Hypoxia	↓SHP1/ERK	Liu et al., 2024
	↓Immune suppression	↓Lactate, ↓TGF-*β*	Liu et al., 2024
Lung cancer	↑Anticancer cytokines	↑INF-*γ*, ↓TNF-*α*, ↑IL-12	Kang et al., 2017
	↓Tumor-promoting cytokines	↓IL-10, ↓IL-4, ↓TGF-*β*	Kang et al., 2017
	↓Invasion/metastasis	↓VEGF, ↓MMP-2, ↓MMP-9, ↓ICAM-1, ↓Akt/c-Myc, ↑miR-342, ↓Cyclin D1, ↑miR-490, ↓DTL, ↓PGE2	Huang and Du, 2020, Kang et al., 2017, Lang et al., 2022, Tao et al., 2020, Zeng et al., 2019, Zhang et al., 2022
	↓Proliferation	↑MEK/ERK1/2	Kang et al., 2017
	↑Apoptosis	↑Caspase-3, ↑Caspase-7, ↑Caspase-9, ↓Bcl-2, ↑Bax	Chen et al., 2016, Zhang et al., 2018
	↑Autophagy	↑PI3K/Akt, ↑MAPK/ERK1/2	Zhang et al., 2018
	Cell cycle arrest	G_0_/G_1_, S	Zeng et al., 2019
	↓Chemoresistance	↑Wnt	Xu et al., 2020
	↓Hypoxia	↓SHP1/ERK	Liu et al., 2024
	↓Immune suppression	↓Lactate, ↓TGF-*β*	Liu et al., 2024
Ovarian cancer	↑Apoptosis	↓PARP1, ↑Bax, ↓Bcl-2, ↑miR125b, ↑Caspase-3	Guo et al., 2016, Ye et al., 2022
	↓Chemoresistance	↓SIRT1, ↓Wnt/*β*-catenin	Duan et al., 2021
	↓Tumor progression	↑PTEN, ↑p53, ↓IL-6, ↓TNF-*α*, ↑c-Jun, ↑Smac	Lin et al., 2024, Ye et al., 2022
Bladder cancer	↓Proliferation	↓mTOR, ↓P70S6K, ↑LAMA3, ↑LAMB3, ↑LAMA4, ↑PAK2, ↑DMD, ↑UTRN, ↓CENPA, ↓Aurora B kinase, ↓PI3K/Akt/mTOR	Ma et al., 2024, Xu et al., 2024
	↑Apoptosis	↓miR-16, ↓NF-*κ*B, ↑Bax, ↓Bcl-2, ↓SIRT1, ↓p53, ↑Caspase-3, ↓cyclin B1, ↓p21	Liu et al., 2018, Xu et al., 2024, Ye et al., 2019
	Cell cycle arrest	G_2_/M	Xu et al., 2024
	↓Tumor progression	↓Circ-0020394, ↑miR-328-3p, ↑NRBP1	Du et al., 2022
	↓Migration/metastasis	↑ITGA1	Ma et al., 2024
Gastrointestinal and colorectal cancers	↑Apoptosis	↓Bcl-2, ↑Bax, ↓c-Myc, ↑Caspase-3, ↑Caspase-9	Yu and Li, 2006, Zhang et al., 2018
	↓Invasion/metastasis	↓MMP-3, ↓MMP-9, ↓VEGF	Zhang et al., 2018
	↓Inflammation	↓TNF-*α*, ↓COX-2, ↓IL-17A, ↓TGF-*β*	Deng et al., 2019
	↓EMT	↓N-cadherin, ↓E-cadherin, ↓Snail, ↓Zeb-1	Deng et al., 2019
	↓Tumor progression	↓TPD1, ↓ALDH1A1, ↓ALDH2, ↓MBNL1, ↓HPGD	Li et al., 2019
	↑Ferroptosis	↑NCOA4	Li et al., 2019
Liver cancer	↑Apoptosis	↑ERK1/2, ↑p38, ↑MAPK, ↑JNK, ↑Caspase-3, ↑Caspase-8, ↑Caspase-9, ↑c-Jun, ↓PI3K/AKT	Zhang et al., 2014, Zhang et al., 2017
	↓EMT	↓Slug, ↓Snail	Zhou et al., 2020
	↓Tumor progression	↑PTEN, ↑miR-21	Zhou, Xue, Wang, & Ren,, 2020; Zhou et al., 2020
Leukemia and Lymphoma	Cell cycle arrest	G_0_/G_1_, S phase	Shao et al., 2010
	↑Apoptosis	↓Bcl-2, ↓Survivin, ↓PML/RAR*α*, ↑JNK, ↑FasL, ↑Caspase-3, ↑Caspase-8, ↑Caspase-9, ↓PARP, ↓cyclin D1, ↑Bax, ↓XIAP, ↓cIAPI, ↓PI3K/Akt, ↓NF-*κ*B	Gan and Yin, 2015, Tang et al., 2010
Neuroblastoma	↑Apoptosis	↑Topoisomerase 1 and 2*α*, ↑Caspase-3, ↑Caspase-9, ↑Bax, ↑Bcl-2, ↑IL-1*β*, ↑TNF*α*, ↑p53, ↓RIRC5	Oltulu et al., 2022, Sui et al., 2024
	↓Invasion	↑TIMP2	Sui et al., 2024
	↑GJIC	↑CX43	Sui et al., 2024
	Disrupting F-actin organization	↓RhoA/ROCK/MLCK	Sui et al., 2024
Cervical cancer	↑Apoptosis	↑Caspase-3, ↑Caspase-9, ↑Bax	Jia et al., 2019
	↓Proliferation	↓PI3K/Akt, ↓mTOR	Jia et al., 2019
Oral squamous cell carcinoma	↓Chemoresistance	↑FBXW7/mTOR	Cai et al., 2023
Pancreatic ductal adenocarcinoma	↓Tumor progression	↓AKT/mTOR	Cai et al., 2023
Prostate cancer	↑Apoptosis	↓Bcl-2, ↑Bax, ↑Caspase-3, ↑Caspase-8, ↑LDH, ↑ROS, ↑Fas, ↓Akt	Li et al., 2021, Pan et al., 2018
	↓Tumor progression	↑Keap1, ↓Nrf2, ↓NQO1	Li et al., 2021
	Inflammation	↑IL-1*β*, ↑IL-6, ↓IL-10	Li et al., 2021

Note: PGE2: prostaglandin E2, PARP1: poly(ADP-ribose) polymerase-1, TPD1: tyrosyl-DNA phosphodiesterase 1, ALDG1A1: aldehyde dehydrogenase 1 family, member A1, NCOA4: nuclear receptor coactivator 4, SHP 1: Src homology region 2 domain-containing phosphatase 1.

### 4.1 PI3K/Akt signaling pathway

The PI3K/Akt pathway is a critical regulator of cell survival, proliferation, and metabolism. Dysregulation, often manifesting as over-expression, of the PI3K/Akt pathway is frequently observed across various cancers, particularly in solid tumors. The aberrant activation of the PI3K/Akt axis plays a critical role in carcinogenesis, cancer cell proliferation, invasion, metastasis, and the development of chemoresistance (Jiang et al., 2020). The PI3K/Akt signaling pathway emerges as a common target of Pue in diverse types, including lymphoma, lung, hepatocellular, and cervical cancers. In lung cancer, Pue has been shown to induce autophagy via PI3K/Akt pathway, thereby inhibiting cancer cell survival and growth (Zhang et al., 2018). Network pharmacology analysis in HCC identified PI3K/Akt as one of the primary targets of Pue (Zhou et al., 2020). Similarly, the modulation of PI3K/Akt signaling by Pue has been observed in cervical cancer and lymphoma (Gan and Yin, 2015, Jia et al., 2019).

### 4.2 MAPK/ERK signaling pathway

Pue exerts its anticancer effects, in part, through the modulation of the MAPK/ERK signaling pathway. In breast cancer, Pue has been shown to disrupt this pathway, leading to reduced cell proliferation, cell cycle arrest, and apoptosis. In lung cancer, Pue promoted autophagy by activating the MAPK/ERK1/2 and PI3K/Akt pathways (Zhang et al., 2018). Pue-mediated increased expression and phosphorylation of ERK1/2, JNK, and p38 in HCC also indicate the involvement of the MAPK pathway. Furthermore, MAPK1 and MAPK3 were identified among the main targets of Pue in HCC via molecular docking studies (Zhang et al., 2017, Zhou et al., 2020).

### 4.3 Caspase-mediated apoptosis

Caspase-mediated apoptosis contributes as a significant anticancer mechanism of Pue across various cancer types, such as breast, lung, ovarian, hepatic, and cervical cancers. Pue primarily induces apoptosis through the activation of Caspases-3, -7, -8, and -9 (Lin et al., 2024, Gan and Yin, 2015, Lin et al., 2009, Tang et al., 2010; Ye, Gao, Fang, Xu, & He, et al., 2022; Yu and Li, 2006, Zhang et al., 2018, Zhang et al., 2014, Zhang et al., 2018).

### 4.4 Inhibition of invasion, metastasis and EMT

Pue’s ability to inhibit cancer cell invasion and metastasis is a recurring observation across various cancers. In breast and lung cancer models, Pue has been shown to reduce the expression of MMPs such as MMP-2, MMP-3, and MMP-9, which are critical enzymes involved in the degradation of the extracellular matrix and subsequent tumor invasion (Huang and Du, 2020, Kang et al., 2017, Lang et al., 2022, Liu et al., 2017, Ma et al., 2024, Tao et al., 2020, Zeng et al., 2019, Zhang et al., 2018, Zhang et al., 2022).

Pue targets the EMT, a crucial process for cancer metastasis, in breast, hepatic, and colorectal cancers by modulating the expression of key EMT-related transcription factors and proteins such as Slu, Snail, Zeb-1, carbonic anhydrase XII, and N- and E-cadherins (Chen et al., 2022, Deng et al., 2019, Zhou et al., 2020).

### 4.5 Cell cycle arrest

Pue’s induction of cell cycle arrest represents a crucial mechanism underlying its anticancer efficacy. Pue has been shown to induce G_2_/M phase arrest in ER-positive and ER-negative breast cancer cells, impairing cell division and proliferation (Lin et al., 2009, Qiu et al., 2022, Zhang et al., 2018). Similarly, leukemia, lung, and bladder cancers exhibit Pue-induced cell cycle arrest, predominantly at the G_0_/G_1_ phase, resulting in decreased cancer cell growth (Jiang et al., 2018, Liu et al., 2018, Shao et al., 2010; Zeng, Shen, Gu, & Wu, 2017).

### 4.6 Tumor immune microenvironment and systemic inflammation

Recent research underscores the critical role of the tumor immune microenvironment in dictating cancer progression and treatment response. This microenvironment, comprising diverse immune cells such as T-cells, macrophages, and dendritic cells, can exert either pro- or anti-tumorigenic effects (Dallavalasa et al., 2021). Pue has demonstrated the capacity to influence immune responses in various contexts, suggesting its potential to modulate the antitumor immune response. Pue has been shown to promote the polarization of TAMs towards the M1 phenotype, thereby enhancing the antitumor immune response (Kang et al., 2017). Furthermore, Pue’s anti-inflammatory effects, mediated through the upregulation of anticancer cytokines such as INF-*γ* and IL-2 could mitigate chronic inflammation, a recognized driver of cancer progression (Kang et al., 2017). By suppressing pro-inflammatory cytokines, including IL-10, IL-4, IL-6, IL-18, and IL-17A, as well as TNF-*α*, TGF-*β*, and COX-2, Pue contributes to reducing the inflammatory milieu that supports tumor growth (Deng et al., 2019, Kang et al., 2017, Li et al., 2021).

### 4.7 Gut microbiota

The gut microbiota has emerged as a significant factor in cancer biology and progression, influencing systemic inflammation, immune responses, and the efficacy of anticancer therapies. Alterations in gut microbiota composition have been linked to the development and progression of various cancers, including colorectal carcinoma (Zhao et al., 2023). Pue has been shown to affect gut microbiota diversity in rat models (Ye et al., 2022). Previous research suggests that Gram-negative bacteria can induce inflammation and elevate the risk of vaginal infection, potentially contributing to carcinogenesis (Sipos et al., 2021). The modulation of the gut microbiota by Pue could potentially enhance the host's immune response against tumors and reduce systemic inflammation. While further research is warranted to fully elucidate this potential mechanism and its contribution to the anticancer activity of Pue, it represents a promising avenue for expanding the therapeutic applications of Pue in cancer treatment.

## 5 Drug delivery systems for Pue in cancer therapy

Extensive research has focused on developing drug delivery systems to enhance the physicochemical and therapeutic properties of Pue by addressing its inherent limitations, including poor water solubility, low bioavailability, and limited targeting. These systems include nanoparticles, nanocrystals, microemulsions, liposomes, dendrimers, and microspheres (Zhang, 2019). The following section explores current drug delivery systems developed for Pue in cancer treatment, highlighting their formulations, mechanisms of action, and therapeutic outcomes. Additionally, Table 3 summarizes drug-delivery systems developed for Pue in cancer treatment and their key outcomes.

**Table 3:** Pue drug delivery systems in cancer treatment.

DDS	Cancer type	Major findings	Specific details	References
Nanoemulsions	Breast cancer	↓ROS, ↓TAFs, ↓collagen deposition, ↑Tumor accumulation, synergism with PTX and PD-L1 blockade, ↑CD8^+^ T cells, ↓immune suppression, ↑survival	112 nm Particle size, 82.4% EE, −5.3 mV Zeta potential, 5-fold ↑ Pue AUC, ↑Pue half-life	Xu et al., 2020
Nanosuspensions	Colon cancer	↓Proliferation, ↓tumor growth, ↑survival	448–481 nm Particle size, PDI 0.19−0.22, 57% tumor inhibition, 100% survival during 3 weeks in mice, ↑Pue tolerability	Wang et al., 2013
	Liver cancer	↑Antitumor activity	218.5 nm Particle size	Lu et al., 2015
Nanoethosomes	Liver cancer	↑Cytotoxicity		Gao et al., 2018
Nanoparticles	Colon carcinoma	↑Cytotoxicity, ↑tumor accumulation, ↓tumor growth	91.8 nm Particle size,	Yi et al., 2019
	Lung cancer	↑Cytotoxicity, ↑apoptosis	70 nm Particle size, pH-responsive release, 84.9% EE, Caspase-3/7-mediated apoptosis	Zhao et al., 2021
Microspheres	Colitis-associated colorectal cancer	↑Colon retention, pH-triggered release, targeted delivery, ↓CAC progression, ↓tumor number, ↓mucosal damage, ↓inflammation, ↓EMT	Dual-loaded PRN-5FU NMs, 81.5 nm particle size	Deng et al., 2019
Hydrogels	Melanoma	Synergism with PTT and gene-targeted therapy, ↓tumor growth, ↓inflammation, ↓EMT	pH-Sensitive alginate, 312.56 µm particle size, 55% Pue release at pH 7.4	Wang et al., 2021
	Breast and lung cancer	↓Hypoxia, ↓immune suppression, synergism with HER 1-CAR-NK cell therapy, ↑CAR-NK cell tumor infiltration, ↑survival, ↓tumor progression	Chitosan-gold Nanorods, NIR-triggered PTT, Pue structural integration	Liu et al., 2024

### 5.1 Nanoemulsions

The study by Xu et al. focused on developing a nanoemulsion formulation of Pue (nanoPue) to enhance its pharmacokinetic profile, tumor-specific accumulation, and therapeutic efficacy in triple-negative breast cancer. NanoPue incorporated soybean lecithin, medium-chain triglycerides, and DSPE-PEG, achieving an encapsulation efficiency (EE) of 82.4%, a particle size of 112 nm, a polydispersity index (PDI) of 0.2, and a zeta potential of − 5.3 mV. For targeting TAFs, the surface of the resulting nanoPue was coated with aminoethyl anisamide (AEAA) as a ligand for the sigma receptor on these cells. The formulation demonstrated sustained drug release (58% cumulative Pue release over 24 h) and reduced ROS production in TGF-*β*-activated NIH3T3 fibroblasts. Pharmacokinetic analysis in mice showed a 5-fold increase in the AUC and 2-fold longer half-life of Pue in the nanoemulsion formulation compared to free Pue. Daily injection of nanoPue into mice bearing the orthotropic 4 T1 breast cancer for 6 d resulted in reduced *α*-SMA-positive TAFs by 6-fold, decreased collagen deposition, and exhibited synergistic effects with paclitaxel and PD-L1 blockade therapy, leading to increased CD8^+^ T-cell infiltration, reducing immunosuppressive cytokines (IL-4, IL-6, IL-10, and IL-13), and increased survival rates. NanoPue injection also reduced lung metastasis to some extent. Safety assessments confirmed no toxicity at doses up to 35 mg/kg, positioning nanoPue as a promising adjuvant for combination therapies (Xu et al., 2020).

### 5.2 Nanosuspensions

Wang et al. (2013) formulated a Pue nanosuspension (Pue-NS) using high-pressure homogenization (HPH) comprising lecithin and hydroxypropyl methylcellulose (HPMC). Pue-NS had a particle size range of 400 − 500 nm and a PDI of 0.1 − 0.2. The treatment of HT-29 colon cancer cells with Pue-NS and Pue solution for 24 h resulted in a concentration-dependent increase in the early apoptosis rate, consequently leading to inhibition of cellular proliferation. However, compared to the Pue solution, the Pue-NS formulation showed a higher early apoptosis induction rate. In HT-29 tumor-bearing mice, Pue-NS exhibited superior antitumor efficacy compared to Pue solution at the same dose. Pue-NS also exhibited lower toxicity, with improved survival rates and tolerability in treated mice. Notably, all mice treated with Pue-NS survived after three weeks, whereas the free Pue group experienced a 10% mortality rate. The enhanced antitumor performance of Pue-NS was attributed to their small particle size, which facilitated passive tumor targeting via the enhanced permeability and retention (EPR) effect (Wang et al., 2013).

Lu et al. developed an oral nanosuspension formulation (Pue-NS) by dispersing Pue in an aqueous solution containing 1% poloxamer 188 (P188), followed by HPH. The resulting Pue-NS exhibited a mean particle size of 218.5 nm, a PDI of 0.4, and a zeta potential of − 18.8 mV. The enhanced solubility of Pue was attributed to the increased surface area of the nanoparticles (Lu et al., 2015). Treatment of HepG2 cells with Pue solution and Pue-NS demonstrated enhanced inhibition of cell growth with IC_50_ values of 5.73 and 3.39 µg/mL, respectively. This improved anticancer activity of Pue-NS was attributed to enhanced cellular uptake and intracellular availability of Pue in the nanosuspension formulation, resulting from reduced particle size and accelerated dissolution rate. Although the study did not include *in vivo* validation, the results suggest that Pue-NS holds promise as a nanoparticle system for improving Pue’s bioavailability and therapeutic efficacy in HCC treatment (Lu et al., 2015).

### 5.3 Nanoethosomes

Gao et al. engineered Pue-loaded nanoethosomes (Pue-NE) using HPH with lecithin, Tween 80, and ethanol. The resulting formulation exhibited a particle size of 91.8 nm, a PDI of 0.1, and a zeta potential of − 6.1 mV (Gao et al., 2018). *In vitro* analysis demonstrated enhanced cytotoxicity of Pue-NE in HepG2 cells (IC_50_: 1.77 µg/mL vs 5.73 µg/mL for free Pue). This improved therapeutic effect was attributed to enhanced cellular uptake and membrane permeability facilitated by the nanoethosome carrier system. However, the lack of *in vivo* efficacy and safety data limits translational implications of this study (Gao et al., 2018).

### 5.4 Nanoparticles

Yi et al. synthesized poly-Pue (PPue) via acryloyl chloride grafting and subsequent polymerization using azobisisobutyronitrile (AIBN). The amphiphilic structure of PPue, resulting from hydrophobic isoflavone/ester groups and hydrophilic glucose moieties, facilitated spontaneous self-assembly into PPue nanoparticles (PPue NPs) with a particle size of 70 nm and a zeta potential of − 39.2 mV (Yi et al., 2019). PPue was optimized as a drug delivery system for colon carcinoma treatment by loading paclitaxel (PTX), yielding PPue@PTX NPs with a drug loading efficiency of 23.8% and an EE of 84.9%. In colon cancer models, PPue@PTX NPs achieved 70% tumor growth inhibition, outperforming free PTX. PPue NPs exhibited no cytotoxicity at concentrations up to 100 µg/mL, while PPue@PTX NPs displayed potent cytotoxicity against CT26 cancer cells, with rapid cellular uptake and intracellular accumulation. The NPs exhibited sustained drug release (80% over 72 h) and Caspase-3/-7-mediated apoptosis. PPue@PTX NPs demonstrated enhanced tumor accumulation, resulting in 70% higher tumor growth inhibition compared to free PTX and reduced systemic toxicity (Yi et al., 2019).

Zhao et al. developed hybrid polymeric nanoparticles (PRN-5FU NMs) co-encapsulating Pue and 5FU using polyethylene glycol-polylactic-co-glycolic acid (PEG-PLGA) via solvent evaporation for targeted lung cancer therapy. These nanoparticles exhibited a mean size of 81.5 nm and a zeta potential of − 7.8 mV. The formulation demonstrated sustained drug release, with 40%–50% of Pue and 5FU released within 24 h, extending to 6 d. *In vitro* evaluations revealed that PRN-5FU NMs significantly enhanced cytotoxicity and apoptosis in A549 and HEL-299 cell lines (Zhao, Liu, Li, Liao, & Ma, 2021). However, the use of HEL-299 cells (normal human embryonic lung fibroblast cell line, ATTC CCL-137 ™) as a cancer model may overstate tumor-specific targeting. The enhanced cytotoxicity observed in HEL-299 cells likely reflects effects on non-cancerous fibroblasts rather than malignant cells, as HEL-299 is not tumorigenic. This misclassification underscores the need for cautious interpretation of their findings, particularly when extrapolating results to lung cancer therapy. Future studies should validate these effects in well-characterized cancer cell lines or *in vivo* models to ensure clinical relevance.

### 5.5 Microspheres

Deng et al. designed pH-sensitive alginate-Pue microspheres as a targeted therapeutic strategy for colitis-associated colorectal cancer (CAC**)** treatment. The microspheres were synthesized through emulsification/internal gelation using sodium alginate, Pue, and nano-calcium carbonate, followed by crosslinking with glutaraldehyde. The Pue microspheres exhibited a particle size of 312.56 µm and a drug loading efficiency of 3%. The formulation demonstrated pH-dependent release kinetics, with 4% of Pue released in acidic gastric fluid (pH 1.2), a moderate 24% release in intestinal fluid (pH 6.8), and a significant 55% release in colonic fluid (pH 7.4). This selective release mechanism minimized premature drug leakage in the upper gastrointestinal tract while enhancing colon-specific delivery. Furthermore, the microspheres demonstrated prolonged colonic retention exceeding 20 h, attributed to alginate’s mucoadhesive properties. *In vivo* evaluations in murine CAC models showed a substantial 50%−70% reduction in tumor burden, inhibited tumor progression, and mitigated mucosal damage. The formulation also downregulated pro-inflammatory cytokines (TNF-*α* and IL-17A) and EMT markers (N-cadherin, Snai1, and MMP-9), highlighting dual anti-inflammatory and anti-metastatic mechanisms (Deng et al., 2019).

### 5.6 Hydrogels

Wang et al. engineered a stimuli-responsive hydrogel (CP@Au@DC_AC50) comprising chitosan, Pue, gold nanorods (GNRs), and the ATOX1 (antioxidant 1 copper chaperone) gene inhibitor, DC_AC50, for combined photothermal therapy (PTT) and gene-targeted therapy in uveal melanoma (UM) treatment and preventing post-injection infections (Wang et al., 2021). Notably, Pue was integrated into this formulation as a structural component of the hydrogel matrix rather than a therapeutic agent. Chitosan-Pue (CP) nanofibers were synthesized using a simple grinding method after mixing with acetic acid solution. CP, GNRs, and DC_AC50 subsequently self-assembled by hydrogen binding, van der Waals forces, hydrophobic, electrostatic, and π-π interactions. The hydrogel demonstrated sustained DC_AC50 release kinetics and photothermal effects under near-infrared (NIR) irradiation. Synergistic antitumor efficacy was achieved through the combined action of PTT and gene therapy: NIR-induced hyperthermia and DC_AC50-mediated suppression of ATOX1 reduced UM cell viability more effectively compared to DC_AC50 or PTT alone. The hydrogel showed 95% tumor regression upon NIR irradiation in orthotopic UM murine models. ATOX1 inhibition further suppressed NF-κB and MAPK signaling pathways, mitigating inflammation and enhancing tumor-specific cytotoxicity. Pue’s structural integration stabilized the hydrogel, while DC_AC50 disrupted redox homeostasis and induced apoptosis in UM cells (Wang et al., 2021).

Liu et al. described an innovative approach for enhancing the effectiveness of chimeric antigen receptor natural killer (CAR-NK) cell therapy using Pue in breast and lung cancer models. The CAR-NK approach combines the specificity of CARs with the broad recognition capabilities of NK cells to target cancer cells. However, the therapeutic effectiveness of CAR-NK is limited due to low tumor penetration and immune suppression. The researchers developed injectable (H_2_O_2_)-responsive hydrogels (puerarin@PEGel) composed of polyethylene glycol dimethacrylate (PEGDMA) mixed with H_2_O_2_ and ferrous chloride. The resulting formulation demonstrated sustained Pue release upon incubation at pH 6.5 and pH 7.4 for 10 d (95% and 85% respectively). Puerarin@PEGel activated the endothelial nitric oxide synthase (eNOS)/nitric oxide (NO)/cyclic guanosine monophosphate (cGMP) signaling axis in tumor endothelial cells, promoting vasodilation, alleviating hypoxia, and inhibiting immunosuppressive lactate and TGF-*β* (Liu et al., 2024). Puerarin@PEGel synergized with HER1-targeted chimeric antigen receptor natural killer (HER1-CAR-NK) cells by reversing tumor immunosuppression: improved oxygenation via eNOS/NO/cGMP enhanced CAR-NK cell infiltration, as well as granzyme B and perforin secretion. Puerarin@PEGel mitigated hypoxia-induced suppression of NK cell activity by blocking the SHP1/ERK signaling pathway. The combination of puerarin@PEGel and HER1-CAR-NK cells significantly suppressed HER1-overexpressing breast tumors, increased survival, and demonstrated no systemic toxicity. This approach highlights vascular normalization as a critical strategy to amplify CAR-NK therapy efficacy in solid tumors by overcoming barriers such as poor immune cell infiltration (Liu et al., 2024).

## 6 Challenges in clinical translation

While preclinical studies have demonstrated promising anticancer effects of Pue across various cancer types (Table 2), several challenges hinder its translation into clinical applications. A primary limitation is the poor oral bioavailability of Pue, attributed to its low water solubility, extensive first-pass metabolism, and limited intestinal permeability (Li et al., 2014, Quan et al., 2007, Tu et al., 2013). In addition, Pue is susceptible to degradation under exposure to light, heat, and oxygen (Liu, Xiang, Li, Hu, & Ye, 2012). These limitations necessitate the development of novel drug delivery systems to enhance Pue's absorption, distribution, and target-specific delivery.

Another challenge lies in the standardization of Pue extracts. Boué et al. reported that kudzu root extract, containing Pue, exhibited estrogenic activity and promoted the growth of estrogen-dependent breast cancer cells (Boue et al., 2003). In contrast, Lin et al. observed that purified Pue inhibited cell growth in both ER-positive and triple-negative breast cancer cell lines (Lin et al., 2009). These inconsistencies highlight the importance of variations in isoflavonoid composition, extraction methods, and the presence of other bioactive compounds in *Pueraria* extracts. As Nimpao demonstrated, the ratio of aglycosides to glycosides, environmental conditions during plant cultivation, and metabolic activation processes can significantly affect Pue's biological activity (Nimpao, 2006). Therefore, standardized extraction protocols and precise quantification of Pue content are crucial for ensuring reproducibility and comparability across studies. Different studies have used varying concentrations of Pue, and since most studies have shown dose-dependent anticancer effects of Pue, conducting dose–response studies for dose optimization is necessary, especially in hormone-dependent cancers such as breast, cervical, ovarian, and prostate cancer.

The long-term safety profile of Pue, especially with chronic administration, remains to be fully elucidated. Additionally, potential drug-drug interactions, particularly in combination therapies, warrant careful evaluation. According to Ye et al., the timing of Pue administration is also crucial for optimal antitumor effects (Ye et al., 2022).

In addition to the factors mentioned above, the complex interactions of Pue with the gut microbiota should also be considered. As Ye et al. observed, Pue can modulate the gut microbiota, which plays a significant role in tumor progression and immunosuppression in cancer. The gut microbiota's influence on Pue's metabolism and its downstream effects on anticancer immunity and drug interactions require further investigation.

Understanding the optimal treatment regimen, including initiation time, duration, and dosage, is essential for clinical translation. The suggestion that Pue induces DNA damage in bladder cancer cells raises concerns about potential long-term effects and genomic instability. Further investigations into the nature of DNA damage, repair mechanisms, and potential mutagenic effects of Pue are essential. Investigating potential off-target effects and unintended consequences of Pue treatment is also necessary. Understanding the impact of Pue on normal cellular functions and possible adverse effects is crucial for its safe and effective use in cancer therapy.

## 7 Discussion

Pue has emerged as a promising multi‑targeted anticancer agent. Extensive studies across a broad spectrum of malignancies, including breast, lung, ovarian, bladder, prostate, hepatic, gastrointestinal, hematological, and other cancers, have consistently demonstrated Pue’s potential to inhibit tumor cell proliferation, induce apoptotic and autophagic cell death, suppress invasion and metastasis, and overcome chemoresistance. Pue demonstrates several advantageous features compared to other anticancer agents, supporting its potential for clinical translation. Its activity spans a wide range of cancer types, suggesting a broad applicability against various malignancies. Pue also exerts its anticancer effects through a multimodal mechanism of action, simultaneously targeting multiple pathways crucial for cancer progression, which can help overcome resistance mechanisms and improve therapeutic efficacy. Notably, some studies indicate that Pue can selectively target cancer cells, potentially reducing toxicity to normal cells, a significant benefit over conventional chemotherapies. Furthermore, Pue has shown promise in enhancing the effectiveness of existing chemotherapeutic drugs and reversing drug resistance, making it a valuable candidate for combination therapies. The ongoing development of novel drug delivery systems is also improving Pue's solubility, bioavailability, and targeted delivery, which further enhances its therapeutic potential and reduces off-target effects. Mechanistically, Pue modulates key signaling pathways such as NF-κB, MAPK/ERK, PI3K/Akt, JAK2/STAT3, and Wnt/*β-*catenin, induces Caspase-mediated apoptosis, cell‑cycle arrest, EMT inhibition, and immune‑microenvironment reprogramming, underscoring its multifaceted mode of action.

Despite Pue’s demonstrated anticancer efficacy across a broad spectrum of malignancies, the body of preclinical and mechanistic work in breast cancer is currently the most extensive, largely owing to its notable capacity to reverse chemoresistance (e.g. to adriamycin and oxaliplatin) and to potentiate combination regimens with both chemotherapeutics and immunotherapies. Pue’s pharmacological effects are highly context-dependent, varying with tumor subtype, treatment modality, and cellular microenvironment, and therefore, robust, head-to-head *in vivo* comparisons and ultimately clinical trials will be required to establish its optimal applications across different cancer types.

Despite these promising preclinical results, clinical translation of Pue is hindered by its poor water solubility, limited bioavailability, rapid metabolism, and lack of standardized formulations. Recent advances in nanotechnology-based formulations, such as nanoemulsions, nanosuspensions, nanoethosomes, and polymeric nanoparticles, have significantly improved Pue’s pharmacokinetics, tumor targeting, and therapeutic efficacy. These novel delivery systems not only enhance Pue’s systemic distribution but also enable co‑delivery with established chemotherapeutics, potentiating synergistic anticancer effects and reducing off‑target toxicity.

Nevertheless, several challenges remain to be addressed before Pue can be used in clinical practice. Standardized extraction, rigorous quality control, and dose-optimization studies are crucial to establish safety and efficacy profiles. Detailed pharmacodynamic and pharmacokinetic investigations in larger animal models and early-phase clinical trials will be critical to determine optimal dosing regimens, tissue distribution, and potential drug–drug interactions. Moreover, a deeper understanding of Pue’s selectivity toward cancer versus normal cells, its immunomodulatory properties, and its long‑term safety is required to mitigate potential adverse effects. Notably, previous findings underscore the dual nature of Pue’s activity in prostate cancer, where Pue’s potent pro-apoptotic effects are accompanied by increased IL-6 and IL-1*β* and reduced IL-10, potentially fostering a pro-tumorigenic inflammatory milieu. This highlights the need for *in vivo* studies to evaluate its net therapeutic effect and supports the rationale for exploring adjunct strategies, such as IL-6 pathway inhibitors or antioxidants, to mitigate adverse immunomodulatory consequences.

These findings align Pue with other natural anticancer agents that exert multifaceted effects on tumor cells. For example, curcumin induces apoptosis and inhibits proliferation by targeting signaling pathways such as NF-κB, STAT3, and PI3K/Akt. Resveratrol modulates Bcl-2 family proteins, suppresses angiogenesis, and inhibits inflammatory mediators such as Cox-2 and TNF-*α*. Similarly, epigallocatechin-3-gallate (EGCG), a major catechin in green tea, promotes cell cycle arrest and apoptosis by interfering with EGFR and VEGF pathways. These agents, similar to Pue, operate through a combination of apoptotic, anti-proliferative, anti-inflammatory, and anti-angiogenic mechanisms, highlighting Pue’s potential as a promising, multi-targeted candidate in anticancer therapy.

Future research should focus on (i) elucidating Pue’s molecular targets within specific tumor subtypes and the tumor microenvironment, including its impact on gut microbiota and systemic inflammation; (ii) optimizing advanced delivery systems for controlled release and active targeting; (iii) exploring rational combination regimens with immunotherapies, targeted agents, and conventional chemotherapies; and (iv) validating biomarkers predictive of Pue responsiveness. Integration of systems biology, network pharmacology, and high‐throughput screening approaches will accelerate the identification of novel Pue analogs and synergistic combinations.

In conclusion, Pue represents a versatile natural compound with significant anticancer potential. By overcoming formulation and bioavailability limitations and rigorously evaluating its mechanistic foundations and clinical safety, Pue could be advanced into the clinic as an effective, low-toxicity adjunct in cancer therapy. Continued multidisciplinary efforts are warranted to translate Pue from bench to bedside and to realize its promise as a component of next‑generation anticancer strategies.

## CRediT authorship contribution statement

**Aida Alizamir:** Writing – original draft, Data curation, Investigation. **Masoud Khanaghaei:** Writing – original draft, Data curation. **Farshad Mirzavi:** Visualization. **Ali Nokhodchi:** Supervision, Writing – review & editing. **Laleh Ghavami:** Writing – original draft. **Bita Taghizadeh:** Conceptualization, Data curation, Project administration, Validation, Writing – original draft, Writing – review & editing.

## Declaration of competing interest

The authors declare that they have no known competing financial interests or personal relationships that could have appeared to influence the work reported in this paper.
